# Survival analysis of patients with primary breast duct carcinoma and lung adenocarcinoma: a population-based study from SEER

**DOI:** 10.1038/s41598-021-94357-4

**Published:** 2021-07-20

**Authors:** Fengyuan Lv, Mingliang Cheng, Liang Jiang, Xiaoping Zhao

**Affiliations:** 1 Center of Stomatology, Tongji Hospital, Tongji Medical College, Huazhong University of Science and Technology, Wuhan, China; 2 Tongji Hospital, Tongji Medical College, Huazhong University of Science and Technology, Wuhan, China

**Keywords:** Cancer epidemiology, Breast cancer, Non-small-cell lung cancer

## Abstract

The appeal to enroll patients with primary breast and lung cancer in clinical trials is increasing, but survival of these two primary cancers remains to be elucidated. This study analyzed the prognosis of primary breast duct carcinoma with subsequent lung adenocarcinoma (BCLA) and primary breast duct carcinoma with prior lung adenocarcinoma (LABC). Cohorts of 3,515 patients with BCLA and 654 patients with LABC were identified from the Surveillance, Epidemiology, and End Results database. Patients were classified into simultaneous two primary cancer (sTPC), metachronous two primary cancer (mTPC1), or mTPC2 groups when the interval times between breast and lung cancer were within 6 months, between 7 and 60 months, or over 60 months, respectively. The propensity score matching program (PSM) was applied to determine the survival of BCLA/LABC relative to single breast/lung cancer. Cox proportional hazard regression model and competing risk modes were performed to identify confounders associated with all-cause and cancer-specific death, respectively. Survival of patients with LABC/BCLA relative to single breast/lung cancer was accessed via median survival time. The survival of patients with BCLA/LABC was generally poor compared with the survival of those with single breast cancer. The PSM-estimated HR in the sTPC group with BCLA and in the mTPC1 and mTPC2 groups with LABC were 0.75 (95% CI 0.62–0.90), 0.52 (95% CI 0.27–0.98), and 0.36 (95% CI 0.20–0.65), respectively, whereas the SHRs were 0.80 (95% CI 0.66–0.97), 0.68 (95% CI 0.34–1.34), and 0.46 (95% CI 0.27–0.80), respectively, compared with those in the single lung cancer group. By contrast, the survival rates of the remaining patients did not differ. The median survival times since secondary malignancy were 42, 23, and 20 months in the sTPC, mTPC1, and mTPC2 groups with BCLA, respectively, and 18, 60, and 180 months in those with LABC, respectively. For patients with BCLA, the adjusted Cox regression suggested incidences of all-cause deaths in mTPC1group were statically higher than those in sTPC group, whereas the incidences of all-cause and cancer-specific death in the mTPC1 and mTPC2 groups were statistically lower than those in the sTPC group. The prognosis of patients with breast cancer and subsequent lung cancer of over 18 months was not significantly different than that of single lung cancer, which supported the profound appeal to increase the involvement of these two primary cancers in potential beneficial clinical trials. For patients with lung cancer and prior breast cancer of within 6 months and subsequent breast cancer of over 18 months, prognosis was improved relative to single lung cancer. Therefore, additional attention is needed to eliminate the potential bias may when these patients are recruited in the clinical trials.

## Introduction

Adenocarcinoma and duct carcinoma are the main histologies of lung and breast malignancies in the United States, respectively^1, 2^. Breast cancer prognosis has been considerably improved, with an approximately 90% 5-year (2011–2017) rate of relative survival. In lung cancer, the prognosis is discouraging because the 5-year (2011–2017) rate of relative survival is less than 20%^3, 4^. With advanced diagnosis and increased life span, the number of patients diagnosed with primary lung adenocarcinoma after/before primary breast duct carcinoma has increased^5^. The two primary cancers are considered simultaneous if the interval between them is within 6 months, whereas others are considered metachronous. Enrolling these patients in clinical trials that may benefit survival has an increased appeal, but the uncertainty of the prior cancer history’s effect on the prognosis causes hesitation in the decision^6–8^. Indeed, accumulating evidences suggest that a cancer may enhance the ability of invasion through fusion and transfer of exosomes between two cancer types^9, 10^. Therefore, the prognosis of patients with simultaneous two primary cancers (sTPCs), metachronous two primary cancers (mTPCs), and single cancer of the same histological type at the same anatomic site may vary. In contrast to most population-based research focusing on the incidence and site of secondary primary cancer (SPC) after breast/lung cancer, the estimates of indicated histology of breast and lung cancers are lacking. Although several clinical observations showed the prognosis of these patients, the conclusions are contradictory^11–14^. Understanding these results is difficult, partly due to the limited scale of patients involved and the different histological types of malignancies in these reports. Another challenge is evaluating the first and secondary primary malignant stages with various criteria.

The prognosis of breast cancer and prior lung cancer may differ from that of breast cancer and subsequent lung cancer. Radiotherapy plays an important role in breast and lung cancer management. However, the risks of side effects, such as radiation pneumonitis and heart disease, from current regular radiation in breast cancer after lung cancer radiation therapy, particularly at the same side, are higher than those in lung cancer management after breast cancer radiotherapy. Moreover, malignancies may share the same characteristics despite having different histological origins. Therefore, targeting drugs for breast malignancy may target lung malignancy, and vice versa^15^.

With data from the Surveillance, Epidemiology, and End Results (SEER) database of the National Cancer Institute, this study was designed to depict the prognosis of two simultaneous and metachronous primary cancers, namely, first primary breast duct carcinoma–secondary primary lung adenocarcinoma (BCLA) and first primary lung adenocarcinoma–secondary primary breast duct carcinoma (LABC), relative to that of single breast/lung cancer. This study also aimed to characterize other clinical and sociodemographic factors associated with survival in patients with primary breast and lung cancers.

## Methods

In accordance with the rule of international multiple primary cancers, records of primary breast duct carcinoma and primary lung and bronchus (lung) adenocarcinoma diagnosed from January 1975 to December 2018 were collected from the SEER database via SEER*Stat software version 8.3.5 (accession number: 12223-Nov2018). Information such as race, sex, insurance and marital status, age at diagnosis, malignant site, malignant histology, surgical status, and radiation were collected. Chemo-treatment was not included because related information was missing in most patients. The International Classification of Diseases for Oncology Site Recode (third edition, ICD-O-3) was applied to identify the anatomical site of malignancy, and SEER historic Stage A was used in classifying the patients’ clinical stages (localize, regional, distant, and unknown). After incomplete medical records and records of only autopsy or death certificate were excluded, 3,515 patients with BCLA and 654 patients with LABC were identified. In addition, 705,725 patients with single breast duct carcinoma and 282,486 patients with lung adenocarcinoma diagnosed from January 1975 to December 2018 were selected (Table S7). The sequence of breast and lung cancers was determined using SEER sequence number as reported^16^. In brief, “00” was assigned to patients with single cancer. For those with two primary cancers, the first and second diagnosed cancers were indicated as “01” and “02,” respectively. The interval time between the diagnosis of first primary cancer (FPC) and SPC was subsequently calculated. FPC and SPC were considered sTPCs and mTPCs if the interval was within 6 months and over 6 months, respectively^17^. To be noted, FPC and SPC were considered mTPC1 if the interval was between 7 and 60 months, whereas mTPC2 if the interval was over 60 months^18^.

Propensity score matching (PSM) program with 1:1 ratio without replacement was performed between patients with two primary cancers and single breast/lung cancer. In brief, the caliper size was set at 0.1, and covariate balance was determined when the absolute standardized difference between matched covariates was within 10% (Table S8–S23). Psestimate command was executed for covariates, such as race, sex, insurance and marital status, year of diagnosis, age, stage, surgical status, and radiation, to achieve the optimal imitative effect^19^. Subsequently, the selected linear or quadratic function of covariates was plugged into the program. For the mTPC1 group with first primary breast/lung cancer, the matched patients with single cancer were required to have a survival time of at least 18 months. For the mTPC2 group, the patients with single cancer were required to have a survival time of at least 60 months.

The year of secondary malignancy diagnosis was classified into three groups to reduce the confounding variables, such as survival time prolonged by medical progression: before 1995, between 1995 and 2005, and after 2005. Overall survival was accessed using median survival time, which was obtained via Kaplan–Meier mode. Variables such as race, sex, first and secondary marital status, and first and secondary insurance status were included in Cox proportional hazard regression model if the *p* value of the indicated variable regression was not more than 0.1. Cause of death was defined based on the International Statistical Classification of Diseases and Related Health Problems (10th Revision)^20^. In brief, events such as breast-, lung-, and liver-related deaths were considered interested ones, whereas events such as septicemia-, heart disease-, and diabetes mellitus-related deaths were considered competing ones. Fine and Gray’s competing risk regression modes were subsequently applied to evaluate the cancer-specific survival with the variables entered in Cox mode. Raw data from SEER were processed on MS Excel (version 2016, Microsoft Corp., LLC) and Stata statistical software (version 15.1, Stata Corp., LLC). All statistical analyses were performed using STATA. p < 0.05 was considered statistically significant.

In compliance with the SEER Research Data Use Agreement, the patients’ privacy was protected, and all methods were carried out in accordance with relevant guidelines and regulations. This study was approved by the Institutional Review Board of Tongji Hospital, Tongji Medical College, Huazhong University of Science and Technology. Because of the retrospective nature of this study, patients’ informed consent was also exempted by the institute.

## Results

### Basic characteristics

A total of 3515 patients with BCLA, 654 patients with LABC, 705,725 patients with single breast duct carcinoma, and 282,486 patients with lung adenocarcinoma were identified. The median ages at secondary diagnosis in the sTPC, mTPC1, and mTPC2 groups with BCLA were 67, 70, and 73 years, respectively, whereas the median ages at secondary diagnosis in the sTPC, mTPC1, and mTPC2 groups with LABC were 69, 67, and 64 years, respectively. In patients with BCLA or LABC, the highest and lowest ratios of breast surgery performed were found in the mTPC2 and sTPC groups, respectively. In patients with BCLA, the highest and lowest ratios of lung surgery performed were found in the sTPC and mTPC2 groups, respectively, whereas the highest and lowest ratios in those with LABC were found in the mTPC2 and sTPC groups, respectively. Other baseline characteristics of the selected patients are shown in Table 1 and Tables S1,S2.

**Table 1:** Summary of identified two primary cancer patients.

	BCLA				LABC			
	All	sTPC	mTPC1	mTPC2	All	sTPC	mTPC1	mTPC2
No	3515	530	1056	1929	654	212	277	165
**Race**								
NHW	2813	418	845	1550	520	167	219	134
NHB	300	48	85	167	77	23	37	17
NHA	219	37	68	114	29	12	11	6
Hispanic	166	26	53	87	24	9	8	7
Others	17	1	5	11	4	1	2	1
**Sex**								
Men	28	5	14	9	8	1	5	2
Women	3487	525	1042	1920	646	211	272	163
**First diagnosis of year**								
> 2005	1102	340	504	258	296	131	137	28
1995–2005	1436	134	379	923	215	52	90	73
< 1995	977	56	173	748	143	29	50	64
**Age of first diagnosis**								
Median	64	67	67	61	67	69	67	64
IQR	55–71	60–75	59–73	53–68	60–74	63–75.5	61–74	57–70
**First marital status**								
Single	1516	275	482	759	322	118	133	71
Married	1884	233	533	1118	303	83	131	89
Unknown	115	22	41	52	29	11	13	5
**First insurance**								
Uninsured	12	5	6	1	5	2	3	0
Insured	952	311	448	193	257	117	117	23
Unknown	2551	214	602	1735	392	93	157	142
**First cancer stage**								
Localized	2408	298	765	1345	258	47	128	83
Regional	951	144	256	551	161	39	79	43
Distant	81	36	28	17	144	96	41	7
Unknown	75	52	7	16	91	30	29	32
**First site**								
	Breast	Breast	Breast	Breast	Lung	Lung	Lung	Lung
**First histology**								
	8500	8500	8500	8500	8140	8140	8140	8140
**First surgery**								
Yes	131	101	19	11	201	129	60	12
No	3371	421	1034	1916	450	83	214	153
Unknown	13	8	3	2	3	0	3	0
**First radiotherapy**								
Yes	11	7	4	0	9	3	6	0
No	2351	353	746	1252	323	109	151	63
Unknown	1153	170	306	677	322	100	120	102
**Interval time**								
Mean	70	2	33	125	23	1	28	100
IQR	25–132	1–3	21–45	90–177	2–62	0–2	16–39	77–140
**Secondary diagnosis of year**								
> 2005	2361	344	624	1393	397	132	164	101
1995–2005	827	131	310	386	169	51	76	42
< 1995	327	55	122	150	88	29	37	22
**Secondary marital status**								
Single	1808	279	506	1023	333	113	138	82
Married	1542	230	496	816	279	85	122	72
Unknown	165	21	54	90	42	14	17	11
**Secondary insurance**								
Uninsured	22	5	4	13	4	2	1	1
Insured	2160	318	568	1274	351	116	145	90
Unknown	1333	207	484	642	299	94	131	74
**Age of Secondary diagnosis**								
Median	71	67	70	73	71	69	69	74
IQR	64–78	60–75	62–76	66–79	64–77	36–79	63–76	67–79
**Secondary histology**								
	8140	8140	8140	8140	8500	8500	8500	8500
**Secondary site**								
	Lung	Lung	Lung	Lung	Breast	Breast	Breast	Breast
**Secondary cancer stage**								
Localized	847	187	276	384	422	146	172	104
Regional	777	135	251	391	114	26	60	28
Distant	1363	128	374	861	49	21	19	9
Unknown	528	80	155	293	69	19	26	24
**Secondary surgery**								
Yes	2007	231	559	1217	134	87	35	12
No	1491	299	489	703	517	123	241	153
Unknown	17	0	8	9	3	2	1	0
**Secondary radiotherapy**								
Yes	34	2	15	17	6	1	2	3
No	1165	187	320	658	286	79	121	86
Unknown	2316	341	721	1254	362	132	154	76
**Major cause of cancer-related death**^**1**^								
Lung	61.51%	49.13%	56.12%	67.58%	44.37%	53.22%	47.46%	20.69%
Breast	20.76%	29.77%	26.08%	15.54%	23.45%	19.82%	20.34%	17.24%
**Major cause of noncancer-related death**^**1**^								
Heart disease	3.61%	4.91%	3.46%	3.38%	9.89%	4.68%	10.17%	19.54%
COPD	2.56%	3.18%	3.09%	2.11%	4.14%	1.75%	4.52%	9.20%

*NHW* Non-Hispanic White, *NHB* Non-Hispanic Black, *NHA* Non-Hispanic Asian or Pacific Islander, 8500: duct carcinoma, 8140: Adenocarcinoma. Major cancer-related death^1^: percent of cancer-related death in total death; Major noncancer-related death^1^: percent of noncancer-related death in total death. *COPD* chronic obstructive pulmonary disease.

### Survival relative to single cancer

After the PSM program was conducted, 688 patients with single breast cancer and BCLA were included in the matched cohort. Survival estimates suggested that the median overall survival times since the secondary malignancy of BCLA and single breast cancer were 115 and 184 months, respectively. Based on interval time, patients with BCLA were categorized into groups (sTPC, mTPC1, and mTPC2), and each categorized group was subsequently matched with single breast cancer. The median overall survival times of the sTPC, mTPC1, and mTPC2 groups were general shorter than those of the matched patients with single breast cancer. When divided by year of diagnosis, the median survival time of categorized sTPC, mTPC1, and mTPC2 groups were general shorter than that of corresponding sing breast cancer (Table 2). Cox analysis suggested that the incidences of all-cause and cancer-specific death in the sTPC, mTPC1, and mTPC2 groups were higher than those in the corresponding patients (Table S3).

**Table 2:** Overall-survival analysis between patients with BCLA and matched single breast/lung cancer.

	All				sTPC				mTPC1				mTPC2			
	NO	50%	95% CI		NO	50%	95% CI		NO	50%	95% CI		NO	50%	95% CI	
**SBC**																
> 2005	335	-	83	-	219	-	66	-	157	-	-	-	150	-	-	-
1995–2005	192	-	-	-	96	178	152	-	114	148	92	-	254	-	-	-
< 1995	145	110	60	251	41	205	106	-	61	102	61	165	224	-	-	-
Overall	672	184	138	-	356	187	152	-	332	167	114	241	628	-	-	-
**BCLA**																
> 2005	348	72	62	92	229	40	27	87	158	57	50	67	158	121	108	-
1995–2005	194	120	98	135	97	38	25	47	115	55	49	63	251	158	145	173
< 1995	145	215	182	251	39	41	15	75	59	67	56	79	219	222	198	244
Overall	687	115	103	127	365	40	29	52	332	60	54	63	628	175	161	184
**SLA**																
> 2005	664	33	27	37	234	24	20	34	394	28	23	36	380	29	25	36
1995–2005	337	17	14	23	101	31	19	41	171	18	15	24	166	22	13	33
< 1995	191	21	14	28	51	30	14	89	91	26	18	44	89	15	10	19
Overall	1192	24	21	30	386	27	22	34	656	24	20	29	635	26	21	30
**BCLA**																
> 2005	723	35	27	44	261	53	30	87	407	25	23	36	402	35	26	47
1995–2005	348	23	18	30	102	39	27	50	175	24	16	31	167	25	18	34
< 1995	176	30	21	39	48	41	19	72	92	29	18	44	91	20	13	34
Overall	1247	29	25	33	411	42	34	54	674	25	22	30	660	29	24	34

*BCLA* breast cancer with subsequent primary lung cancer; *SBC* Single breast cancer; *SLA* Single lung cancer, − not reached; Overall survival was determined by median survival time (month).

A total of 1,306 patients with single lung adenocarcinoma and BCLA were selected using another PSM program. The median overall survival times of single lung adenocarcinoma and BCLA were 24 and 29 months, respectively. When stratified by interval time, the median overall survival times in the sTPC, mTPC1, and mTPC2 groups with BCLA were 42, 25, and 29 months, respectively. In the matched patients with single lung cancer, the median overall survival times were 27, 24, and 26 months, respectively. When classified by year of diagnosis, median survival of both sTPC and mTPC2 groups diagnosed after 2005 was statically longer than that of matched single lung cancer (Table 2). The incidences of all-cause and cancer-specific deaths in the sTPC group were statistically lower (sTPC group: all-cause death rate, 0.75; 95% CI 0.62–0.90; cancer-specific death rate, 0.80; 95% CI 0.66–0.97) than those in patients with single lung cancer. In the mTPC1 and mTPC2 groups, the incidences of all-cause and cancer-specific death did not statistically differ (Table S4).

The prognosis of breast cancer and prior lung cancer may differ from that of breast cancer and subsequent lung cancer. A total of 274 patients with single lung adenocarcinoma and LABC were included in the matched cohort. The median overall survival times of patients with secondary cancer and single lung cancer with LABC were 34 and 99 months, respectively. In the sTPC, mTPC1, and mTPC2 groups with LABC, the median overall survival times were 20, 151, and 335 months, respectively; in the corresponding patients, the median overall survival times were 27, 40, and 257 months, respectively. When stratified by year of diagnosis, median survival time of both mTPC1 and mTPC2 groups diagnosis between 1995 and 2005 were longer than that of matched single lung cancer (Table 3). The incidences of all-cause deaths in the mTPC1 and mTPC2 groups were statistically lower than those in the corresponding patients (mTPC1: 0.52; 95% CI 0.27–0.98; mTPC2: 0.36; 95% CI 0.20–0.65), and cancer-specific deaths in mTPC2 groups was lower than matched group. In the sTPC group, the incidences of all-cause and cancer-specific deaths did not statistically differ (Table S5).

**Table 3:** Overall-survival analysis between patients with LABC and matched single breast/lung cancer.

	All				sTPC				mTPC1				mTPC2			
	NO	50%	95% CI		NO	50%	95% CI		NO	50%	95% CI		NO	50%	95% CI	
**SLA**																
> 2005	184	75	31	–	86	29	18	34	84	40	34	116	20	–	–	–
1995–2005	52	18	11	39	43	18	9	47	18	34	25	83	46	–	–	–
< 1995	11	26	4	–	20	17	8	102	–	–	–	–	36	216	114	320
Overall	247	34	25	53	149	27	17	32	102	40	34	83	102	257	165	–
**LABC**																
> 2005	141	55	37	93	96	19	12	28	56	85	54	–	22	–	–	–
1995–2005	83	154	77	–	40	12	6	26	33	151	47	–	47	–	–	–
< 1995	46	169	75	335	22	30	8	71	13	173	25	–	33	335	176	–
Overall	270	99	59	155	158	20	12	27	102	151	59	–	102	335	240	–
**SBC**																
> 2005	180	–	54	–	147	114	82	–	105	68	47	–	84	88	39	–
1995–2005	82	–	–	–	25	–	70	–	54	178	143	–	33	–	87	–
< 1995	52	139	60	–	5	67	9	–	28	–	106	–	22	–	38	–
Overall	314	–	139	–	177	–	82	–	187	–	117	–	139	–	–	–
**LABC**																
> 2005	184	39	23	91	104	17	10	25	107	46	18	–	87	–	83	–
1995–2005	80	37	24	127	46	10	5	19	54	51	27	–	33	180	83	–
< 1995	50	32	25	66	22	32	7	87	26	60	12	90	21	100	30	–
Overall	314	36	27	54	172	17	10	23	187	47	27	90	141	180	100	–

*LABC* breast cancer with prior primary lung cancer; *SBC* Single breast cancer; *SLA* Single lung cancer, – not reached; Overall survival was determined by median survival time (month).

For patients with single breast cancer and LABC in the matched cohort, the median overall survival times in the sTPC, mTPC1, and mTPC2 groups were 17, 47, and 180 months, respectively, whereas the median overall survival times of the corresponding breast cancer patients were -, -, and – months, respectively. When stratified by year of diagnosis, median survival time of classified sTPC, mTPC1, and mTPC2 groups were shorted than that of matched single breast cancer (Table 4). The incidence of all-cause and cancer-specific deaths in the sTPC and mTPC1 groups increased compared with that in single breast cancer. In the mTPC2 group, the difference between the incidence of all-cause death and that of cancer-specific death was not significant (Table S6).

**Table 4:** Overall-survival analysis since secondary diagnosis in patients with BCLA/LABC.

	BCLA				LABC			
	NO	50%	95% CI		NO	50%	95% CI	
**sTPC**								
First cancer stage								
Localize	290	41	31	49	46	48	28	65
Regional	144	89	56	105	39	32	18	87
Distant	35	14	5	29	90	8	5	10
Unknown	49	19	8	33	25	26	9	44
Subtotal	518	42	34	53	200	18	12	25
**mTPC1**								
First cancer stage								
Localize	721	22	20	26	126	169	60	–
Regional	243	25	20	31	78	60	27	139
Distant	28	25	12	37	41	17	8	32
Unknown	6	11	1	–	26	49	14	68
Subtotal	998	23	20	26	271	60	35	96
**mTPC2**								
First cancer stage								
Localize	1242	20	18	22	81	180	83	–
Regional	503	21	17	24	42	164	164	–
Distant	17	14	8	50	7	–	2	–
Unknown	14	18	5	46	32	100	30	–
Subtotal	1776	20	18	22	162	180	100	–
Overall	3292	23	21	25	633	52	39	68

Group sTPC, mTPC1 and mTPC2 were categorized by first cancer stage; Overall survival was determined by median survival time (month).

– not reached.

### Survival between synchronous and metachronous two primary cancers

**Figure 1:**
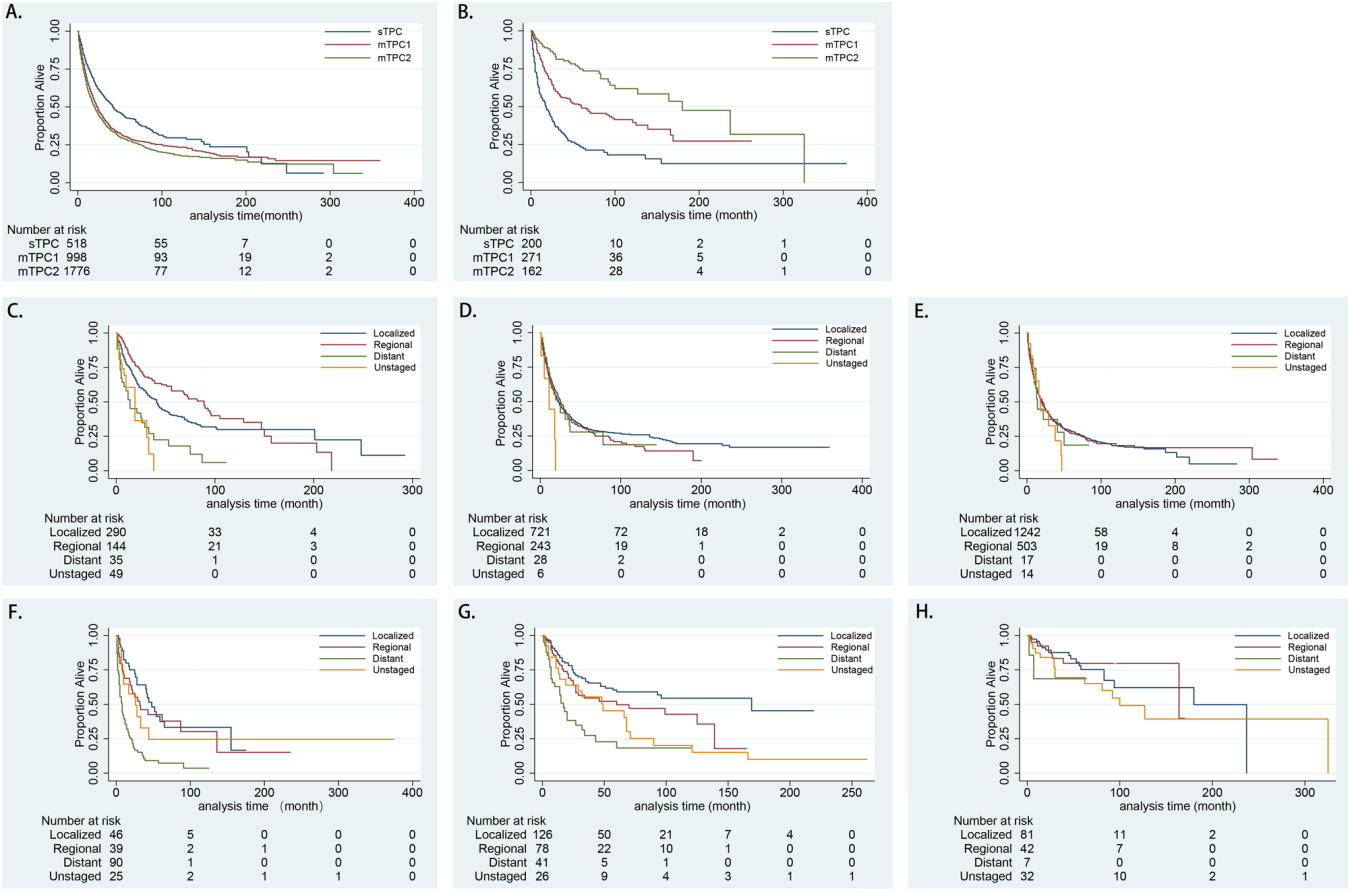
(**A**) Survival estimates of BCLA since secondary diagnosis stratified by group sTPC, mTPC1 and mTPC2; (**B**) Survival estimates of LABC since secondary diagnosis stratified by group sTPC, mTPC1 and mTPC2; (**C**–**E**) Survival estimates of BCLA in respective group sTPC, mTPC1 and mTPC2 since secondary diagnosis stratified by breast cancer stage; (**F**–**H**) Survival estimates of LABC in respective group sTPC, mTPC1 and mTPC2 since secondary diagnosis stratified by breast cancer stage.

**Figure 2:**
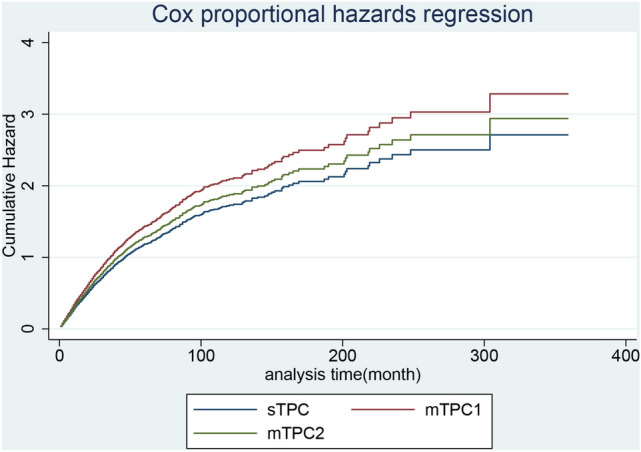
For breast cancer with subsequent lung cancer, adjusted cumulative hazard of all-cause mortality since secondary cancer was classified into group sTPC, mTPC1 and mTPC2.

**Figure 3:**
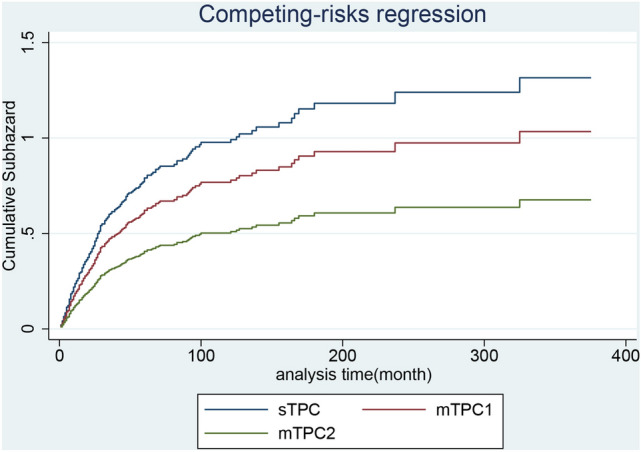
For breast cancer with subsequent lung cancer, adjusted cumulative hazard of cancer-specific mortality since secondary cancer was classified into group sTPC, mTPC1 and mTPC2.

Among the primary causes of death in patients with primary breast and lung cancer, lung cancer was the leading cause of cancer-related death in patients with BCLA or LABC, whereas heart disease and chronic obstructive pulmonary disease accounted for most noncancerous deaths (Table 1). In patients with BCLA, the median overall survival since secondary diagnosis in the sTPC, mTPC1, and mTC2 groups were 42, 23, and 20 months, respectively and estimated survival was displayed in Fig. 1. When divided by first cancer stage, median survival time of ‘regional stage’ was statically longer than that of ‘localized stage’, and ‘distant stage’ was statically shorter than ‘localized stage’ in sTPC group. For mTPC1 and mTPC2 groups, there was no statical difference between ‘regional stage’ and ‘localized stage’, as well as ‘distant stage’ and ‘localized stage’. (Table 4). Adjusted for the first malignant stage, age at secondary diagnosis, and secondary surgery status, the incidences of all-cause deaths in the mTPC1 group was statically higher than sTPC group, whereas incidences of all-cause deaths in the mTPC2 group was not statistically different from those in the sTPC group (Figs. 2 and 3). In particular, secondary diagnosis age over 75 (versus secondary diagnosis age less than 55), breast malignancy at the distant stage (versus localized), year of secondary diagnosis between 1955 and 2005 and before 1995 (versus over 2005), lung malignancy at the regional stage (versus localized), and lung malignancy at the distant stage (versus localized) were remarkably associated with increased all-cause and cancer-specific deaths. Insured at first diagnosis (versus uninsured) and lung surgery performed (versus not performed) were remarkably associated with decreased all-cause and cancer-specific deaths (Table 5).

**Table 5:** Covariates associated with all-cause and cancer-specific death in patients with BCLA.

	H. R	P	95% CI		SHR	P	95% CI	
**Group**								
sTPC	1.00				1.00			
mTPC1	1.21	0.01	1.04	1.41	1.14	0.07	0.99	1.31
mTPC2	1.08	0.33	0.92	1.28	1.00	0.99	0.86	1.16
**Category**								
> 2005	1.00				1.00			
1995–2005	1.22	0.07	0.99	1.50	1.18	0.13	0.95	1.47
< 1995	1.55	< 0.01	1.22	1.98	1.47	< 0.01	1.14	1.90
**Race**								
NHW	1.00				1.00			
NHB	1.01	0.95	0.86	1.18	0.97	0.72	0.82	1.14
NHA	0.77	0.01	0.63	0.94	0.83	0.06	0.69	1.01
Hispanic	1.13	0.29	0.91	1.40	1.02	0.86	0.79	1.32
Others	0.96	0.90	0.51	1.80	1.09	0.76	0.63	1.88
**Sex**								
Male	1.00				1.00			
Female	0.72	0.18	0.44	1.17	0.95	0.86	0.53	1.69
**Insurance1**								
Uninsure	1.00				1.00			
Insure	0.54	0.11	0.25	1.14	0.51	0.04	0.27	0.96
Unknown	0.62	0.21	0.29	1.31	0.60	0.12	0.32	1.14
**Marital1**								
Single	1.00				1.00			
Married	0.84	0.01	0.74	0.96	0.89	0.09	0.78	1.02
Unknown	0.87	0.29	0.67	1.13	0.84	0.18	0.65	1.09
**Seerstage1**								
Localized	1.00				1.00			
Regional	1.01	0.77	0.92	1.12	1.00	0.95	0.90	1.11
Distant	1.38	0.03	1.04	1.82	1.34	0.04	1.01	1.79
Unknown	1.28	0.19	0.89	1.85	1.18	0.38	0.81	1.70
**Surgery1**								
No	1.00				1.00			
Yes	0.91	0.47	0.70	1.18	1.09	0.56	0.82	1.43
Unknown	1.83	0.08	0.94	3.55	2.20	0.01	1.26	3.86
**Radiation1**								
No	1.00				1.00			
Yes	0.92	0.87	0.34	2.49	0.85	0.66	0.41	1.76
Unknown	0.99	0.99	0.36	2.70	0.91	0.80	0.44	1.89
**Age**								
< 55	1.00				1.00			
55–75	1.26	0.01	1.06	1.49	1.19	0.05	1.00	1.41
> 75	1.59	< 0.01	1.32	1.91	1.37	< 0.01	1.13	1.65
**Marital2**								
Single	1.00				1.00			
Married	1.03	0.67	0.90	1.18	1.02	0.81	0.88	1.17
Unknown	0.86	0.19	0.69	1.08	0.91	0.39	0.74	1.12
**Insurance2**								
Uninsure	1.00				1.00			
Insure	0.74	0.37	0.39	1.42	0.69	0.20	0.39	1.22
Unknown	0.74	0.38	0.38	1.45	0.70	0.24	0.38	1.27
**Seerstage2**								
Localized	1.00				1.00			
Regional	2.08	< 0.01	1.81	2.40	1.98	< 0.01	1.74	2.26
Distant	4.15	< 0.01	3.61	4.77	3.77	< 0.01	3.29	4.32
Unknown	2.24	< 0.01	1.87	2.69	2.08	< 0.01	1.75	2.48
**Surgery2**								
No	1.00				1.00			
Yes	0.45	< 0.01	0.40	0.51	0.52	< 0.01	0.46	0.58
Unknown	0.65	0.24	0.32	1.33	0.69	0.49	0.24	1.98
**Radiation2**								
No	1.00				1.00			
Yes	0.80	0.32	0.51	1.25	0.77	0.29	0.48	1.25
Unknown	0.77	0.24	0.49	1.20	0.74	0.22	0.46	1.19

All variables were used in multivariate analysis.

*Insurance1* first insurance status; *Marital1* first marital status; *Seerstage1* first cancer stage; *Surgery1* first surgery; *Radiation1* first radiation; *Insurance2* secondary insurance status; *Marital2* secondary marital status; *Seerstage2* secondary cancer stage; *Surgery2* secondary surgery; *Radiation2* secondary radiation; *H.R* risk of all-cause death; *SHR* risk of cancer-specific death.

**Figure 4:**
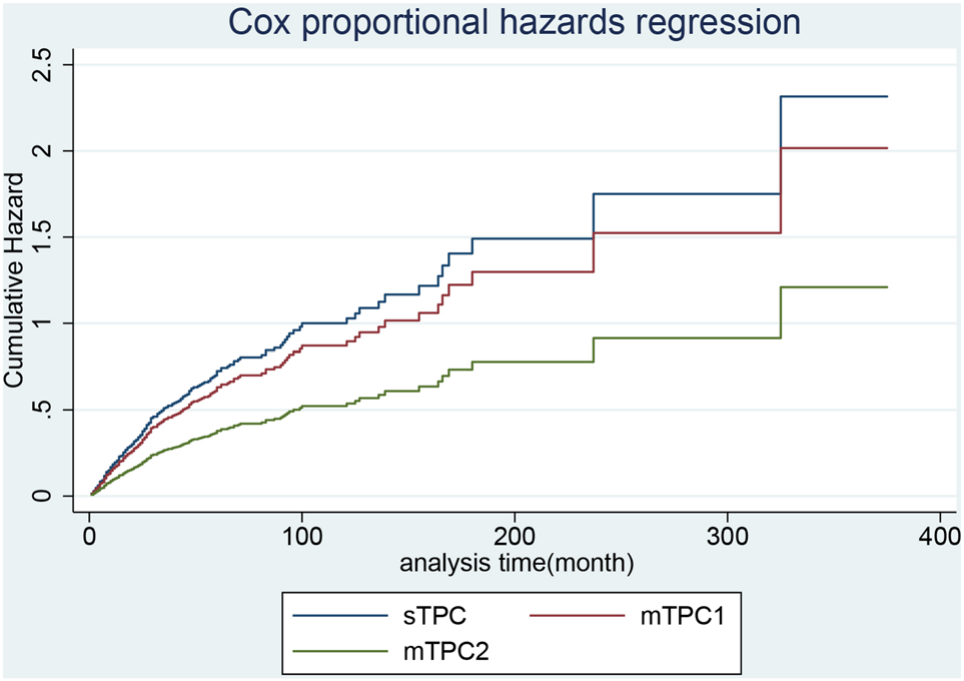
For breast cancer with prior lung cancer, adjusted cumulative hazard of all-cause mortality since secondary cancer was classified into group sTPC, mTPC1 and mTPC2.

**Figure 5:**
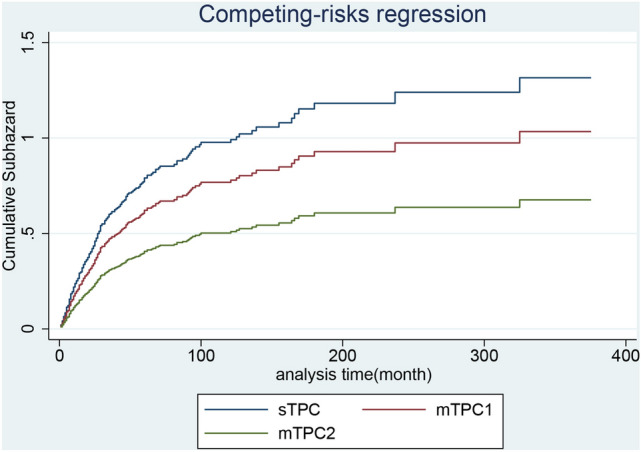
For breast cancer with prior lung cancer, adjusted cumulative hazard of cancer-specific mortality since secondary cancer was classified into group sTPC, mTPC1 and mTPC2.

In patients with LABC, the median overall survival since secondary diagnosis in the sTPC, mTPC1, and mTC2 groups were 18, 60, and 180 months, respectively and displayed in Fig. 1. When divided by first cancer stage, median survival time of ‘regional and distant’ stage were statically shorter than that of ‘localized stage’ in the sTPC, mTPC1, and mTC2 groups (Table 4). Adjusted for the first malignant stage, first surgery status, and secondary surgery status, the incidence of all-cause death since secondary diagnosis in the mTPC2 group was remarkably lower than that in the sTPC group, and the incidence of cancer-specific death in the mTPC1 and mTPC2 groups was lower than that in the sTPC group (Figs. 4 and 5). In detail, lung adenocarcinoma at the distant stage (versus localized) and year of secondary diagnosis before 1995 (versus over 2005) were remarkably associated with increased all-cause and cancer-specific deaths, whereas breast and lung surgery performed (versus not performed) was remarkably associated with decreased all-cause and cancer-specific deaths (Table 6).

**Table 6:** Covariates associated with all-cause and cancer-specific death in patients with LABC.

	H. R	P	95% CI		SHR	P	95% CI	
**Group**								
sTPC	1.00				1.00			
mTPC1	0.87	0.34	0.66	1.15	0.79	0.10	0.59	1.05
mTPC2	0.52	< 0.01	0.34	0.80	0.51	< 0.01	0.34	0.77
**Category**								
> 2005	1.00				1.00			
1995–2005	1.63	0.02	1.09	2.44	1.51	0.04	1.01	2.24
< 1995	2.63	< 0.01	1.51	4.60	2.84	< 0.01	1.66	4.85
**Race**								
NHW	1.00				1.00			
NHB	1.01	0.98	0.67	1.50	0.79	0.35	0.49	1.29
NHA	0.76	0.36	0.42	1.37	0.86	0.61	0.49	1.52
Hispanic	0.65	0.19	0.34	1.24	0.67	0.20	0.36	1.23
Others	< 0.01	1.00	< 0.01	-	< 0.01	< 0.01	< 0.01	< 0.01
**Age**								
< 55	1.00				1.00			
55–75	1.10	0.64	0.73	1.68	0.90	0.64	0.58	1.40
> 75	1.23	0.40	0.76	1.99	0.86	0.52	0.53	1.38
**Insurance1**								
Uninsure	1.00				1.00			
Insure	0.97	0.97	0.27	3.54	0.65	0.57	0.15	2.82
Unknown	0.79	0.73	0.21	2.97	0.52	0.38	0.12	2.28
**Seerstage1**								
Localized	1.00				1.00			
Regional	1.25	0.18	0.90	1.72	1.22	0.21	0.89	1.67
Distant	1.93	< 0.01	1.35	2.76	1.84	< 0.01	1.30	2.61
Unknown	0.97	0.91	0.59	1.59	0.91	0.70	0.57	1.45
**Marital1**								
Single	1.00				1.00			
Married	0.93	0.58	0.73	1.19	0.97	0.83	0.75	1.26
Unknown	0.63	0.12	0.35	1.12	0.75	0.34	0.42	1.35
**Surgery1**								
No	1.00				1.00			
Yes	0.48	< 0.01	0.34	0.67	0.55	< 0.01	0.39	0.78
Unknown	0.74	0.77	0.10	5.66	0.67	0.74	0.06	7.26
**Radiation1**								
No	1.00				1.00			
Yes	1.31	0.70	0.32	5.39	1.32	0.59	0.48	3.58
Unknown	1.19	0.81	0.29	4.95	1.11	0.84	0.40	3.10
**Seerstage2**								
Localized	1.00				1.00			
Regional	1.21	0.22	0.89	1.66	1.25	0.14	0.93	1.68
Distant	2.52	< 0.01	1.72	3.70	2.46	< 0.01	1.53	3.97
Unknown	1.73	0.07	0.95	3.13	1.28	0.44	0.68	2.42
**Surgery2**								
No	1.00				1.00			
Yes	0.57	< 0.01	0.40	0.80	0.59	< 0.01	0.41	0.84
Unknown	2.48	0.14	0.75	8.27	2.63	< 0.01	1.41	4.88
**Radiation2**								
No	1.00				1.00			
Yes	0.64	0.54	0.15	2.66	0.70	0.66	0.14	3.39
Unknown	0.91	0.89	0.22	3.76	0.96	0.96	0.20	4.63

All variables were used in multivariate analysis.

*Insurance1* first insurance status; *Seerstage1* first cancer stage; *Surgery1* first surgery; *Radiation1* first radiation; *Seerstage2* secondary cancer stage; *Surgery2* secondary surgery; *Radiation2* secondary radiation; *H.R* risk of all-cause death; *SHR* risk of cancer-specific death.

## Discussion

In this study, the survival of patients with primary breast and lung cancers was analyzed. The results suggested that the survival rate of patients with BCLA was generally poorer than that of patients with matched single breast cancer but not inferior to that of patients with single lung cancer. In addition, the primary causes of cancer- and noncancer-related deaths in patients with BCLA were identical to those in patients with single lung cancer. For patients with LABC, the survival since lung cancer was poorer than that in corresponding single breast cancer. The survival of patients with simultaneous LABC was similar to that of patients with single lung cancer, whereas the survival of patients with metachronous LABC was generally improved. To the knowledge of the authors, this study was the largest population-based study of survival in primary breast duct carcinoma and lung adenocarcinoma.

Several studies depicted the survival of patients with lung cancer and prior cancer. Prior head and neck cancer history was reported to be associated with compromised survival of lung cancer patients, whereas gastrointestinal tract and colorectal cancer history were not relevant to the prognosis^21–23^. These results supported the argument from another report that the prognosis of lung cancer with prior cancer was heterogenous among types and stages^24^. For lung cancer and prior breast malignancy, Zhou et al. reported that patients with prior breast cancer generally experienced survival inferior to that of patients without prior cancer^16^. Laccetti et al. and Martin et al. revealed that patients with lung cancer and prior breast cancer generally had no worse survival compared with patients without prior cancer history^7, 25^, and a synchronous breast malignancy may be associated with improved survival^26^. These results are conflicting; the higher number of patients involved in Laccetti’s report (patients diagnosed between 2004 and 2008 versus 1992 and 2009) and the bias involved in PSM program may be part of the underlying reasons. In the present study, entire records with a complete history of breast and lung cancers linked in the SEER database were included, and potential biases, such as medical progress accompanying time of treatment, were excluded at the beginning of the PSM program. The results suggested that the overall and cancer-specific survival of patients with lung cancer and prior breast cancer (interval time between two cancers of over 18 months) was generally similar to those without prior cancer. However, more studies are required because these patients with lung cancer (interval time of less than 6 months) may have a survival advantage, which may interfere with the trial results.

Information about the prognosis of lung cancer with subsequent breast cancer is lacking, which could be considerably attributed to its rareness. Zhou et al. reported that patients with prior lung cancer experienced survival similar to those without prior cancer. Caijin Lin et al. demonstrated that patients with prior cancer experienced worse overall survival and improved cancer-specific survival^27^. Both studies were based on limited clinical observations. The present study suggested that the survival of metachronous LABC since secondary cancer was improved relative to that of synchronous LABC and single lung cancer. Potential explanations included an advantageous cancer survivor phenotype in patients with metachronous LABC and more frequent, intensive care-associated lead- and length–time biases^8, 28, 29^. Another possible reason was the unexpected effect of drugs targeting hormonal pathways in breast cancer treatment. Estrogen receptor α is highly expressed in women suffering from lung adenocarcinoma, which is associated with poor prognosis^30–32^. In-vitro experiments and animals suggested that targeting estrogen receptor signaling leads to proliferation inhibition and increased response to chemotherapy in lung adenocarcinoma^31, 33^. Finally, inappropriate treatment may be referred to synchronous two primary cancers due to the misjudged stage. For patients with synchronous primary breast and lung cancers, chemoradiotherapy may be referred due to the difficulty in distinguishing lung cancer invasion-related satellite nodules from breast cancer metastasis. However, the survival rate of T4 satellite lung cancer treated with radical resection was better than that of patients treated with chemoradiotherapy^34^. These hypothesizes may partly explain the survival advantage of patients with simultaneous BCLA and metachronous LABC. However, further studies are still required to reveal the underlying mechanisms.

The limitation of this study included several dimensions. First, the information on chemotherapy, targeted therapy, and other covariates associated with lung cancer prognosis was inaccessible, whereas that on radiotherapy and insurance was limited in the SEER database, which may affect the results of PSM and Cox analysis. Second, the information on patients’ psychological status was missing. The ratios of lung surgery performed in the sTPC group were higher than those in the mTPC group for BCLA but lower for LABC, suggesting that the number of patients inclined to abandon treatment varied among groups. Third, given the large scale of patients included in the SEER database, the number of patients qualified in this study was still low, particularly those categorized for analysis. Patients from other countries are necessary for further study. Finally, bias was inevitable due to the properties of a prospective epidemiological study.

In conclusion, this study depicted the prognosis of patients with BCLA and LABC. Compared with patients with single breast cancer, those with BCLA and LABC generally experienced poor survival. Patients with metachronous LABC and synchronous BCLA experienced better prognosis than those with single lung cancer, and the survival of the remaining patients statistically differed. Although the surgery for breast and lung cancer was associated with improved survival, its rate was lower in the sTPC group. Further study is required to clarify the mechanisms mediating survival advantage in BCLA/LABC relative to single lung cancer and increase the rate of surgery performed for breast and lung cancers.

## Supplementary Information

Supplementary Information.
