# Author Correction: Survival analysis of patients with primary breast duct carcinoma and lung adenocarcinoma: a population-based study from SEER

**DOI:** 10.1038/s41598-021-97687-5

**Published:** 2021-09-01

**Authors:** Fengyuan Lv, Mingliang Cheng, Liang Jiang, Xiaoping Zhao

**Affiliations:** 1 Center of Stomatology, Tongji Hospital, Tongji Medical College, Huazhong University of Science and Technology, Wuhan, China; 2 Tongji Hospital, Tongji Medical College, Huazhong University of Science and Technology, Wuhan, China

Correction to: *Scientific Reports* https://doi.org/10.1038/s41598-021-94357-4, published online 20 July 2021

In the original version of this Article Fengyuan Lv and Mingliang Cheng were omitted as equally contributing authors. The original Article has been corrected.

