# Supplementary material for: Survival analysis of patients with primary breast duct carcinoma and lung adenocarcinoma: a population-based study from SEER
Source: Sci Rep. 2021 Jul 20;11:14790. doi: 10.1038/s41598-021-94357-4 (PMC8292419; doi:10.1038/s41598-021-94357-4)
Supplement: Supplementary file 1 — Supplementary Information. [file 41598_2021_94357_MOESM1_ESM.docx]

Tab S1 Overall survival analysis of single breast cancer, LABC and BCLA since breast cancer (month)

|  |  | Survival of LABC since breast cancer | | | | Survival of BCLA since breast cancer | | | |  | Single breast cancer | | | |
| --- | --- | --- | --- | --- | --- | --- | --- | --- | --- | --- | --- | --- | --- | --- |
| Year of diagnosis | | Subjects | 50% | 95% CI | | Subjects | 50% | 95% CI | |  | Subjects | 50% | 95% CI | |
|  | >2005 | 385 | 58 | 41 | - | 1101 | 85 | 78 | 90 |  | 359179 | - | - | - |
|  | 1995-2005 | 164 | 37 | 27 | 83 | 1434 | 135 | 129 | 142 |  | 210494 | - | - | - |
|  | <1995 | 84 | 60 | 29 | 81 | 975 | 194 | 182 | 209 |  | 127717 | 447 | 431 | 461 |
|  | Total | 633 | 52 | 39 | 68 | 3510 | 136 | 131 | 143 |  | 697390 | - | - | - |

BCLA: breast duct carcinoma with subsequent primary lung adenocarcinoma; LABC: breast duct carcinoma with prior primary lung adenocarcinoma; Overall survival was determined by median survival time (month); -: not reached

Tab S2 Overall survival analysis of single lung cancer, LABC and BCLA since lung cancer (month)

|  |  | Survival of LABC since lung cancer | | | | Survival of BCLA since lung cancer | | | | Single lung cancer | | | |
| --- | --- | --- | --- | --- | --- | --- | --- | --- | --- | --- | --- | --- | --- |
| Year of diagnosis | | Subjects | 50% | 95% CI | | Subjects | 50% | 95% CI | | Subjects | 50% | 95% CI | |
|  | >2005 | 294 | 63 | 47 | 101 | 2197 | 24 | 22 | 27 | 119974 | 14 | 14 | 15 |
|  | 1995-2005 | 213 | 177 | 105 | 236 | 785 | 21 | 18 | 24 | 68876 | 10 | 10 | 11 |
|  | <1995 | 142 | 169 | 117 | 232 | 310 | 23 | 19 | 31 | 45614 | 9 | 9 | 9 |
|  | Total | 649 | 128 | 95 | 155 | 3292 | 23 | 21 | 25 | 234464 | 12 | 12 | 12 |

BCLA: breast duct carcinoma with subsequent primary lung adenocarcinoma; LABC: breast duct carcinoma with prior primary lung adenocarcinoma; Overall survival was determined by median survival time (month);

Tab S3 All cause and cancer-specific death between BCLA and matched single breast cancer

|  |  | sTPC | | | | | | mTPC1 | | | | | | mTPC2 | | | | | |
| --- | --- | --- | --- | --- | --- | --- | --- | --- | --- | --- | --- | --- | --- | --- | --- | --- | --- | --- | --- |
|  |  | H. R | 95% CI | | SHR | 95% CI | | H. R | 95% CI | | SHR | 95% CI | | H. R | 95% CI | | SHR | 95% CI | |
| Group | |  |  |  |  |  |  |  |  |  |  |  |  |  |  |  |  |  |  |
|  | SBC | 1.00 |  |  | 1.00 |  |  | 1.00 |  |  | 1.00 |  |  | 1.00 |  |  | 1.00 |  |  |
|  | BCLA | 3.19 | 2.46 | 4.12 | 2.94 | 2.27 | 3.81 | 2.48 | 1.97 | 3.11 | 2.33 | 1.85 | 2.93 | 2.22 | 1.83 | 2.71 | 2.59 | 2.10 | 3.20 |
| Race | |  |  |  |  |  |  |  |  |  |  |  |  |  |  |  |  |  |  |
|  | NHW | 1.00 |  |  | 1.00 |  |  | 1.00 |  |  | 1.00 |  |  | 1.00 |  |  | 1.00 |  |  |
|  | NHB | 0.83 | 0.55 | 1.24 | 0.74 | 0.48 | 1.15 | 1.42 | 1.04 | 1.93 | 1.36 | 1.00 | 1.84 | 1.06 | 0.85 | 1.34 | 1.02 | 0.81 | 1.27 |
|  | NHA | 0.77 | 0.49 | 1.20 | 0.81 | 0.52 | 1.26 | 0.98 | 0.68 | 1.41 | 1.00 | 0.70 | 1.44 | 0.59 | 0.45 | 0.79 | 0.64 | 0.50 | 0.84 |
|  | Hispanic | 0.70 | 0.41 | 1.20 | 0.70 | 0.41 | 1.18 | 1.23 | 0.87 | 1.73 | 1.17 | 0.80 | 1.69 | 0.95 | 0.69 | 1.30 | 0.98 | 0.74 | 1.29 |
|  | Others | 0.99 | 0.13 | 7.52 | 0.85 | 0.34 | 2.15 | 2.94 | 1.33 | 6.47 | 2.87 | 1.59 | 5.18 | 0.68 | 0.28 | 1.66 | 0.68 | 0.31 | 1.47 |
| Sex | |  |  |  |  |  |  |  |  |  |  |  |  |  |  |  |  |  |  |
|  | Men | 1.00 |  |  | 1.00 |  |  | 1.00 |  |  | 1.00 |  |  | 1.00 |  |  | 1.00 |  |  |
|  | Women | 0.73 | 0.18 | 3.03 | 0.82 | 0.20 | 3.39 | 0.53 | 0.29 | 0.98 | 0.79 | 0.41 | 1.53 | 1.42 | 0.63 | 3.19 | 1.77 | 0.75 | 4.17 |
| Marital | |  |  |  |  |  |  |  |  |  |  |  |  |  |  |  |  |  |  |
|  | Single | 1.00 |  |  | 1.00 |  |  | 1.00 |  |  | 1.00 |  |  | 1.00 |  |  | 1.00 |  |  |
|  | Married | 0.88 | 0.68 | 1.13 | 0.90 | 0.70 | 1.15 | 0.84 | 0.68 | 1.05 | 0.89 | 0.71 | 1.11 | 1.00 | 0.84 | 1.20 | 1.04 | 0.87 | 1.23 |
|  | Unknown | 1.27 | 0.80 | 2.02 | 1.29 | 0.85 | 1.96 | 0.95 | 0.49 | 1.84 | 0.91 | 0.47 | 1.76 | 0.83 | 0.56 | 1.24 | 0.78 | 0.53 | 1.16 |
| Insurance | |  |  |  |  |  |  |  |  |  |  |  |  |  |  |  |  |  |  |
|  | Uninsured | 1.00 |  |  | 1.00 |  |  | 1.00 |  |  | 1.00 |  |  | 1.00 |  |  | 1.00 |  |  |
|  | Insured | 0.50 | 0.20 | 1.25 | 0.45 | 0.17 | 1.17 | 0.37 | 0.11 | 1.24 | 0.44 | 0.18 | 1.06 | 0.04 | 0.01 | 0.30 | 0.04 | 0.01 | 0.16 |
|  | Unknown | 0.47 | 0.17 | 1.29 | 0.47 | 0.17 | 1.32 | 0.32 | 0.09 | 1.09 | 0.38 | 0.15 | 0.96 | 0.02 | <0.01 | 0.19 | 0.02 | 0.01 | 0.10 |
| Seerstage | |  |  |  |  |  |  |  |  |  |  |  |  |  |  |  |  |  |  |
|  | Localized | 1.00 |  |  | 1.00 |  |  |  |  |  |  |  |  |  |  |  |  |  |  |
|  | Regional | 1.39 | 1.08 | 1.81 | 1.30 | 1.00 | 1.70 | 1.00 |  |  | 1.00 |  |  | 1.00 |  |  | 1.00 |  |  |
|  | Distant | 1.39 | 0.92 | 2.11 | 1.34 | 0.88 | 2.03 | 1.98 | 1.57 | 2.49 | 1.82 | 1.44 | 2.29 | 1.32 | 1.10 | 1.59 | 1.25 | 1.04 | 1.50 |
|  | Unknown | 1.91 | 0.98 | 3.74 | 2.05 | 1.09 | 3.86 | 1.30 | 0.84 | 2.01 | 1.39 | 0.93 | 2.06 | 1.60 | 0.95 | 2.71 | 1.54 | 0.95 | 2.49 |
| Surgery | |  |  |  |  |  |  |  |  |  |  |  |  |  |  |  |  |  |  |
|  | Yes | 1.00 |  |  | 1.00 |  |  | 1.00 |  |  | 1.00 |  |  | 1.00 |  |  | 1.00 |  |  |
|  | No | 0.41 | 0.30 | 0.57 | 0.52 | 0.38 | 0.72 | 0.67 | 0.39 | 1.16 | 0.74 | 0.41 | 1.32 | 0.96 | 0.47 | 1.99 | 1.18 | 0.57 | 2.42 |
|  | Unknown | 1.25 | 0.59 | 2.68 | 1.58 | 0.73 | 3.43 | 1.37 | 0.18 | 10.74 | 1.42 | 0.10 | 19.76 | 2.41 | 0.49 | 11.93 | 3.01 | 1.32 | 6.87 |
| Radiation | |  |  |  |  |  |  |  |  |  |  |  |  |  |  |  |  |  |  |
|  | No | 1.00 |  |  | 1.00 |  |  | 1.00 |  |  | 1.00 |  |  | 1.00 |  |  | 1.00 |  |  |
|  | Unknown | 1.10 | 0.86 | 1.41 | 1.11 | 0.87 | 1.42 | 1.21 | 0.95 | 1.55 | 1.14 | 0.89 | 1.47 | 1.02 | 0.85 | 1.22 | 1.02 | 0.86 | 1.22 |
| Category | |  |  |  |  |  |  |  |  |  |  |  |  |  |  |  |  |  |  |
|  | >2005 | 1.00 |  |  | 1.00 |  |  | 1.00 |  |  | 1.00 |  |  | 1.00 |  |  | 1.00 |  |  |
|  | 1995-2005 | 1.36 | 0.82 | 2.25 | 1.27 | 0.78 | 2.09 | 1.63 | 1.07 | 2.49 | 1.66 | 1.13 | 2.46 | 1.12 | 0.75 | 1.68 | 1.23 | 0.81 | 1.86 |
|  | <1995 | 1.52 | 0.85 | 2.71 | 1.43 | 0.81 | 2.53 | 1.69 | 1.07 | 2.69 | 1.78 | 1.16 | 2.73 | 0.80 | 0.52 | 1.22 | 1.06 | 0.69 | 1.62 |
| Age | | 1.00 | 0.99 | 1.01 | 0.99 | 0.98 | 1.00 | 1.01 | 1.00 | 1.02 | 1.00 | 1.00 | 1.01 | 1.03 | 1.03 | 1.04 | 1.02 | 1.01 | 1.02 |

BCLA: breast duct carcinoma with subsequent primary lung adenocarcinoma; SBC: single breast cancer; H.R: risk of all-cause death; SHR: risk of cancer-specific death; NHW: Non-Hispanic White; NHB: Non-Hispanic Black; NHA: Non-Hispanic Asian or Pacific Islander; All variables were used in multivariate analysis.

Tab S4 All cause and cancer-specific death between BCLA and matched single lung cancer

|  |  | sTPC | | | | | | mTPC1 | | | | | | mTPC2 | | | | | |
| --- | --- | --- | --- | --- | --- | --- | --- | --- | --- | --- | --- | --- | --- | --- | --- | --- | --- | --- | --- |
|  |  | H. R | 95% CI | | SHR | 95% CI | | H. R | 95% CI | | SHR | 95% CI | | H. R | 95% CI | | SHR | 95% CI | |
| Group | |  |  |  |  |  |  |  |  |  |  |  |  |  |  |  |  |  |  |
|  | SLC | 1.00 |  |  | 1.00 |  |  | 1.00 |  |  | 1.00 |  |  | 1.00 |  |  | 1.00 |  |  |
|  | BCLA | 0.75 | 0.62 | 0.90 | 0.80 | 0.66 | 0.97 | 1.01 | 0.88 | 1.16 | 0.98 | 0.85 | 1.13 | 0.90 | 0.78 | 1.04 | 0.90 | 0.77 | 1.04 |
| Race | |  |  |  |  |  |  |  |  |  |  |  |  |  |  |  |  |  |  |
|  | NHW | 1.00 |  |  | 1.00 |  |  | 1.00 |  |  | 1.00 |  |  | 1.00 |  |  | 1.00 |  |  |
|  | NHB | 0.90 | 0.65 | 1.24 | 0.74 | 0.52 | 1.06 | 1.32 | 1.06 | 1.65 | 1.27 | 1.03 | 1.57 | 1.03 | 0.84 | 1.27 | 1.07 | 0.86 | 1.32 |
|  | NHA | 0.90 | 0.62 | 1.30 | 1.00 | 0.68 | 1.47 | 0.91 | 0.68 | 1.23 | 0.93 | 0.69 | 1.24 | 0.82 | 0.65 | 1.03 | 0.87 | 0.69 | 1.09 |
|  | Hispanic | 0.64 | 0.41 | 1.01 | 0.65 | 0.43 | 0.98 | 1.05 | 0.78 | 1.42 | 0.97 | 0.71 | 1.33 | 1.27 | 0.97 | 1.67 | 1.13 | 0.85 | 1.50 |
|  | Others | 1.21 | 0.16 | 9.28 | 1.31 | 0.74 | 2.31 | 2.39 | 1.05 | 5.43 | 2.66 | 1.09 | 6.51 | <0.01 | . | . | <0.01 | <0.01 | <0.01 |
| Sex | |  |  |  |  |  |  |  |  |  |  |  |  |  |  |  |  |  |  |
|  | Men | 1.00 |  |  | 1.00 |  |  | 1.00 |  |  | 1.00 |  |  | 1.00 |  |  | 1.00 |  |  |
|  | Women | 0.62 | 0.19 | 1.97 | 0.57 | 0.13 | 2.56 | 0.61 | 0.39 | 0.97 | 0.62 | 0.43 | 0.90 | 0.81 | 0.40 | 1.65 | 1.18 | 0.58 | 2.37 |
| Marital | |  |  |  |  |  |  |  |  |  |  |  |  |  |  |  |  |  |  |
|  | Single | 1.00 |  |  | 1.00 |  |  | 1.00 |  |  | 1.00 |  |  | 1.00 |  |  | 1.00 |  |  |
|  | Married | 0.77 | 0.63 | 0.94 | 0.80 | 0.65 | 0.98 | 0.74 | 0.64 | 0.86 | 0.77 | 0.66 | 0.89 | 1.02 | 0.87 | 1.20 | 1.11 | 0.94 | 1.30 |
|  | Unknown | 0.94 | 0.57 | 1.52 | 1.01 | 0.66 | 1.53 | 0.92 | 0.68 | 1.23 | 1.02 | 0.77 | 1.35 | 1.04 | 0.79 | 1.35 | 1.07 | 0.83 | 1.38 |
| Insurance | |  |  |  |  |  |  |  |  |  |  |  |  |  |  |  |  |  |  |
|  | Uninsured | 1.00 |  |  | 1.00 |  |  | 1.00 |  |  | 1.00 |  |  | 1.00 |  |  | 1.00 |  |  |
|  | Insured | 0.47 | 0.20 | 1.08 | 0.41 | 0.15 | 1.11 | 1.42 | 0.35 | 5.74 | 1.41 | 0.39 | 5.12 | 0.83 | 0.34 | 2.02 | 0.84 | 0.33 | 2.17 |
|  | Unknown | 0.41 | 0.16 | 1.04 | 0.36 | 0.13 | 1.06 | 1.67 | 0.41 | 6.87 | 1.66 | 0.45 | 6.14 | 0.99 | 0.40 | 2.48 | 1.04 | 0.39 | 2.74 |
| Seerstage | |  |  |  |  |  |  |  |  |  |  |  |  |  |  |  |  |  |  |
|  | Localized | 1.00 |  |  | 1.00 |  |  | 1.00 |  |  | 1.00 |  |  | 1.00 |  |  | 1.00 |  |  |
|  | Regional | 3.13 | 2.45 | 3.98 | 3.16 | 2.51 | 3.98 | 2.34 | 1.93 | 2.84 | 2.41 | 2.00 | 2.91 | 2.43 | 1.98 | 2.98 | 2.25 | 1.85 | 2.73 |
|  | Distant | 2.06 | 1.55 | 2.75 | 2.06 | 1.51 | 2.79 | 2.12 | 1.73 | 2.60 | 2.22 | 1.81 | 2.73 | 2.35 | 1.89 | 2.92 | 2.26 | 1.83 | 2.79 |
|  | Unknown | 1.73 | 1.16 | 2.59 | 1.81 | 1.21 | 2.72 | 1.81 | 1.36 | 2.42 | 1.86 | 1.39 | 2.47 | 1.55 | 1.15 | 2.10 | 1.49 | 1.13 | 1.97 |
| Surgery | |  |  |  |  |  |  |  |  |  |  |  |  |  |  |  |  |  |  |
|  | Yes | 1.00 |  |  | 1.00 |  |  | 1.00 |  |  | 1.00 |  |  | 1.00 |  |  | 1.00 |  |  |
|  | No | 0.32 | 0.25 | 0.41 | 0.39 | 0.31 | 0.49 | 0.39 | 0.33 | 0.46 | 0.44 | 0.37 | 0.52 | 0.48 | 0.40 | 0.58 | 0.56 | 0.47 | 0.67 |
|  | Unknown |  |  |  |  |  |  | 0.68 | 0.28 | 1.66 | 0.76 | 0.20 | 2.87 | 1.48 | 0.65 | 3.37 | 1.55 | 0.94 | 2.57 |
| Radiation | |  |  |  |  |  |  |  |  |  |  |  |  |  |  |  |  |  |  |
|  | Yes |  |  |  |  |  |  | 1.00 |  |  | 1.00 |  |  | 1.00 |  |  | 1.00 |  |  |
|  | No | 1.00 |  |  | 1.00 |  |  |  |  |  |  |  |  | 2.82E+07 | . | . | 3.50E+06 | 4.57E+05 | 2.68E+07 |
|  | Unknown | 0.81 | 0.67 | 0.98 | 0.80 | 0.66 | 0.97 | 0.97 | 0.84 | 1.12 | 0.97 | 0.84 | 1.12 | 2.58E+07 | 2.21E+07 | 3.01E+07 | 3.13E+06 | 4.10E+05 | 2.39E+07 |
| Category | |  |  |  |  |  |  |  |  |  |  |  |  |  |  |  |  |  |  |
|  | >2005 | 1.00 |  |  | 1.00 |  |  | 1.00 |  |  | 1.00 |  |  | 1.00 |  |  | 1.00 |  |  |
|  | 1995-2005 | 1.54 | 0.95 | 2.49 | 1.44 | 0.91 | 2.29 | 1.15 | 0.86 | 1.54 | 1.21 | 0.90 | 1.61 | 1.01 | 0.78 | 1.32 | 0.99 | 0.75 | 1.29 |
|  | <1995 | 1.72 | 1.01 | 2.92 | 1.60 | 0.97 | 2.64 | 1.38 | 0.97 | 1.95 | 1.39 | 0.97 | 2.00 | 1.52 | 1.13 | 2.03 | 1.44 | 1.07 | 1.94 |
| Age | | 1.01 | 1.00 | 1.02 | 1.00 | 0.99 | 1.01 | 1.01 | 1.01 | 1.00 | 1.01 | 1.00 | 1.00 | 1.01 | 1.01 | 1.02 | 1.01 | 1.00 | 1.02 |

BCLA: breast duct carcinoma with subsequent primary lung adenocarcinoma; SLC: single lung cancer; H.R: risk of all-cause death; SHR: risk of cancer-specific death; NHW: Non-Hispanic White; NHB: Non-Hispanic Black; NHA: Non-Hispanic Asian or Pacific Islander; All variables were used in multivariate analysis.

Tab S5 All cause and cancer-specific death between LABC and matched single lung cancer

|  |  | sTPC | | | | | | mTPC1 | | | | | | mTPC2 | | | | | |
| --- | --- | --- | --- | --- | --- | --- | --- | --- | --- | --- | --- | --- | --- | --- | --- | --- | --- | --- | --- |
|  |  | H. R | 95% CI | | SHR | 95% CI | | H. R | 95% CI | | SHR | 95% CI | | H. R | 95% CI | | SHR | 95% CI | |
| Group | |  |  |  |  |  |  |  |  |  |  |  |  |  |  |  |  |  |  |
|  | SLC | 1.00 |  |  | 1.00 |  |  | 1.00 |  |  | 1.00 |  |  | 1.00 |  |  | 1.00 |  |  |
|  | LABC | 1.17 | 0.87 | 1.56 | 1.24 | 0.93 | 1.66 | 0.52 | 0.27 | 0.98 | 0.68 | 0.34 | 1.34 | 0.36 | 0.20 | 0.65 | 0.46 | 0.27 | 0.80 |
| Race | |  |  |  |  |  |  |  |  |  |  |  |  |  |  |  |  |  |  |
|  | NHW | 1.00 |  |  | 1.00 |  |  | 1.00 |  |  | 1.00 |  |  | 1.00 |  |  | 1.00 |  |  |
|  | NHB | 1.16 | 0.66 | 2.05 | 1.01 | 0.53 | 1.91 | 1.03 | 0.56 | 1.87 | 0.98 | 0.54 | 1.79 | 0.16 | 0.04 | 0.72 | 0.18 | 0.04 | 0.81 |
|  | NHA | 0.47 | 0.25 | 0.88 | 0.53 | 0.31 | 0.92 | 0.94 | 0.47 | 1.89 | 1.02 | 0.55 | 1.87 | 0.27 | 0.04 | 2.11 | 0.34 | 0.04 | 2.71 |
|  | Hispanic | 0.80 | 0.43 | 1.51 | 0.68 | 0.38 | 1.21 | 0.72 | 0.29 | 1.82 | 0.73 | 0.26 | 2.02 | 0.21 | 0.02 | 1.89 | 0.37 | 0.13 | 1.00 |
|  | Others | <0.01 | . | . | <0.01 | <0.01 | <0.01 | 0.76 | 0.10 | 6.10 | 0.92 | 0.10 | 8.23 |  |  |  |  |  |  |
| Sex | |  |  |  |  |  |  |  |  |  |  |  |  |  |  |  |  |  |  |
|  | Men | 1.00 |  |  | 1.00 |  |  | 1.00 |  |  | 1.00 |  |  | 1.00 |  |  | 1.00 |  |  |
|  | Women | 2.17 | 0.30 | 15.91 | 4.13 | 0.79 | 21.52 | 0.66 | 0.27 | 1.62 | 0.66 | 0.23 | 1.91 | 1.34 | 0.10 | 17.54 | 1.85 | 0.21 | 16.73 |
| Marital | |  |  |  |  |  |  |  |  |  |  |  |  |  |  |  |  |  |  |
|  | Single | 1.00 |  |  | 1.00 |  |  | 1.00 |  |  | 1.00 |  |  | 1.00 |  |  | 1.00 |  |  |
|  | Married | 0.94 | 0.70 | 1.26 | 0.93 | 0.70 | 1.23 | 0.85 | 0.52 | 1.38 | 0.90 | 0.57 | 1.43 | 0.47 | 0.25 | 0.87 | 0.54 | 0.29 | 1.00 |
|  | Unknown | 0.37 | 0.19 | 0.73 | 0.45 | 0.22 | 0.92 | 0.93 | 0.38 | 2.31 | 1.18 | 0.54 | 2.55 | 0.98 | 0.20 | 4.72 | 1.17 | 0.38 | 3.59 |
| Insurance | |  |  |  |  |  |  |  |  |  |  |  |  |  |  |  |  |  |  |
|  | Uninsured | 1.00 |  |  | 1.00 |  |  | 1.00 |  |  | 1.00 |  |  | 1.00 |  |  | 1.00 |  |  |
|  | Insured | 0.81 | 0.17 | 3.73 | 0.66 | 0.17 | 2.57 | 0.49 | 0.13 | 1.83 | 0.45 | 0.14 | 1.48 | 9.41 | 0.92 | 96.22 | 13.09 | 1.36 | 126.37 |
|  | Unknown | 1.18 | 0.23 | 5.92 | 1.02 | 0.24 | 4.40 | 0.37 | 0.09 | 1.62 | 0.38 | 0.10 | 1.44 |  |  |  |  |  |  |
| Seerstage | |  |  |  |  |  |  |  |  |  |  |  |  |  |  |  |  |  |  |
|  | Localized | 1.00 |  |  | 1.00 |  |  | 1.00 |  |  | 1.00 |  |  | 1.00 |  |  | 1.00 |  |  |
|  | Regional | 2.05 | 1.30 | 3.23 | 1.89 | 1.18 | 3.04 | 1.73 | 1.01 | 2.95 | 1.82 | 1.10 | 3.02 | 1.00 | 0.44 | 2.27 | 0.75 | 0.31 | 1.81 |
|  | Distant | 2.17 | 1.42 | 3.31 | 2.25 | 1.54 | 3.28 | 2.71 | 1.11 | 6.60 | 1.84 | 0.72 | 4.73 | 3.89 | 1.07 | 14.17 | 2.19 | 0.85 | 5.62 |
|  | Unknown | 1.14 | 0.59 | 2.21 | 0.94 | 0.51 | 1.75 | 1.79 | 0.50 | 6.49 | 2.26 | 0.70 | 7.26 | 1.08 | 0.46 | 2.54 | 1.39 | 0.63 | 3.06 |
| Surgery | |  |  |  |  |  |  |  |  |  |  |  |  |  |  |  |  |  |  |
|  | No | 1.00 |  |  | 1.00 |  |  | 1.00 |  |  | 1.00 |  |  | 1.00 |  |  | 1.00 |  |  |
|  | Yes | 0.30 | 0.20 | 0.45 | 0.35 | 0.23 | 0.53 | 0.41 | 0.23 | 0.73 | 0.44 | 0.25 | 0.77 | 1.15 | 0.32 | 4.18 | 1.24 | 0.32 | 4.80 |
|  | Unknown |  |  |  |  |  |  | 1.27 | 0.15 | 10.69 | 1.74 | 0.78 | 3.89 |  |  |  |  |  |  |
| Radiation | |  |  |  |  |  |  |  |  |  |  |  |  |  |  |  |  |  |  |
|  | Yes | 1.00 |  |  | 1.00 |  |  | 1.00 |  |  | 1.00 |  |  | 1.00 |  |  | 1.00 |  |  |
|  | Unknown | 0.94 | 0.69 | 1.27 | 0.91 | 0.68 | 1.23 | 0.98 | 0.61 | 1.58 | 0.90 | 0.56 | 1.42 | 1.49 | 0.73 | 3.02 | 1.47 | 0.74 | 2.92 |
| Category | |  |  |  |  |  |  |  |  |  |  |  |  |  |  |  |  |  |  |
|  | >2005 | 1.00 |  |  | 1.00 |  |  | 1.00 |  |  | 1.00 |  |  | 1.00 |  |  | 1.00 |  |  |
|  | 1995-2005 | 0.72 | 0.39 | 1.31 | 0.64 | 0.34 | 1.20 | 1.62 | 0.78 | 3.38 | 1.33 | 0.67 | 2.64 | 0.18 | 0.03 | 1.22 | 0.14 | 0.03 | 0.75 |
|  | <1995 | 1.29 | 0.64 | 2.61 | 1.42 | 0.72 | 2.80 | 1.23 | 0.35 | 4.35 | 0.95 | 0.26 | 3.46 | 0.15 | 0.02 | 1.10 | 0.13 | 0.02 | 0.68 |
| Age | | 0.99 | 0.97 | 1.00 | 0.98 | 0.97 | 1.00 | 1.02 | 1.00 | 1.04 | 1.01 | 0.99 | 1.03 | 0.99 | 0.95 | 1.02 | 0.96 | 0.94 | 0.99 |

LABC: breast duct carcinoma with prior primary lung adenocarcinoma; SLC: single lung cancer; H.R: risk of all-cause death; SHR: risk of cancer-specific death; NHW: Non-Hispanic White; NHB: Non-Hispanic Black; NHA: Non-Hispanic Asian or Pacific Islander; All variables were used in multivariate analysis.

Tab S6 All cause and cancer-specific death between LABC and matched single breast cancer

|  |  | sTPC | | | | | | mTPC1 | | | | | | | mTPC2 | | | | |
| --- | --- | --- | --- | --- | --- | --- | --- | --- | --- | --- | --- | --- | --- | --- | --- | --- | --- | --- | --- |
|  |  | H. R | 95% CI | | SHR | 95% CI | | H. R | 95% CI | | SHR | 95% CI | | H. R | 95% CI | | SHR | 95% CI | |
| Group | |  |  |  |  |  |  |  |  |  |  |  |  |  |  |  |  |  |  |
|  | SBC | 1.00 |  |  | 1.00 |  |  | 1.00 |  |  | 1.00 |  |  | 1.00 |  |  | 1.00 |  |  |
|  | LABC | 4.98 | 3.44 | 7.20 | 4.80 | 3.35 | 6.87 | 3.03 | 2.05 | 4.46 | 2.56 | 1.70 | 3.86 | 1.14 | 0.69 | 1.90 | 1.10 | 0.63 | 1.92 |
| Race | |  |  |  |  |  |  |  |  |  |  |  |  |  |  |  |  |  |  |
|  | NHW | 1.00 |  |  | 1.00 |  |  | 1.00 |  |  | 1.00 |  |  | 1.00 |  |  | 1.00 |  |  |
|  | NHB | 1.81 | 1.13 | 2.89 | 1.70 | 1.09 | 2.66 | 0.89 | 0.54 | 1.49 | 0.77 | 0.45 | 1.31 | 0.82 | 0.37 | 1.85 | 0.83 | 0.37 | 1.88 |
|  | NHA | 0.61 | 0.30 | 1.25 | 0.66 | 0.35 | 1.25 | 0.98 | 0.43 | 2.23 | 1.20 | 0.58 | 2.49 | 1.57 | 0.41 | 6.03 | 1.56 | 0.46 | 5.28 |
|  | Hispanic | 0.45 | 0.18 | 1.12 | 0.46 | 0.20 | 1.06 | 0.53 | 0.21 | 1.34 | 0.71 | 0.27 | 1.88 | 0.48 | 0.06 | 3.86 | 0.54 | 0.09 | 3.42 |
|  | Others |  |  |  |  |  |  | 0.55 | 0.13 | 2.37 | 0.71 | 0.13 | 3.87 | <0.01 | . | . | <0.01 | <0.01 | <0.01 |
| Sex | |  |  |  |  |  |  |  |  |  |  |  |  |  |  |  |  |  |  |
|  | Men | 1.00 |  |  | 1.00 |  |  | 1.00 |  |  | 1.00 |  |  | 1.00 |  |  | 1.00 |  |  |
|  | Women | 4.2E+14 | . | . | 2.8E+05 | 3.5E+04 | 2.2E+06 | 0.60 | 0.21 | 1.70 | 0.71 | 0.24 | 2.11 | 4.6E+15 | . | . | 1.2E+07 | 1.9E+06 | 7.3E+07 |
| Marital | |  |  |  |  |  |  |  |  |  |  |  |  |  |  |  |  |  |  |
|  | Single | 1.00 |  |  | 1.00 |  |  | 1.00 |  |  | 1.00 |  |  | 1.00 |  |  | 1.00 |  |  |
|  | Married | 0.88 | 0.62 | 1.24 | 0.93 | 0.66 | 1.32 | 0.77 | 0.53 | 1.11 | 0.79 | 0.53 | 1.18 | 0.70 | 0.39 | 1.25 | 0.84 | 0.47 | 1.51 |
|  | Unknown | 0.59 | 0.29 | 1.19 | 0.63 | 0.31 | 1.29 | 0.73 | 0.27 | 2.02 | 0.56 | 0.17 | 1.88 | 1.82 | 0.74 | 4.48 | 1.52 | 0.65 | 3.57 |
| Insurance | |  |  |  |  |  |  |  |  |  |  |  |  |  |  |  |  |  |  |
|  | Uninsured | 1.00 |  |  | 1.00 |  |  | 1.00 |  |  | 1.00 |  |  | 1.00 |  |  | 1.00 |  |  |
|  | Insured | 1.31 | 0.27 | 6.30 | 1.14 | 0.23 | 5.53 | 0.22 | 0.03 | 1.70 | 0.21 | 0.11 | 0.39 | 0.55 | 0.06 | 5.13 | 0.67 | 0.21 | 2.22 |
|  | Unknown | 1.71 | 0.36 | 8.22 | 1.73 | 0.37 | 8.08 | 0.24 | 0.03 | 2.19 | 0.25 | 0.09 | 0.74 | 1.06 | 0.09 | 12.70 | 1.59 | 0.32 | 7.91 |
| Seerstage | |  |  |  |  |  |  |  |  |  |  |  |  |  |  |  |  |  |  |
|  | Localized | 1.00 |  |  | 1.00 |  |  | 1.00 |  |  | 1.00 |  |  | 1.00 |  |  | 1.00 |  |  |
|  | Regional | 1.89 | 1.21 | 2.95 | 1.96 | 1.25 | 3.08 | 1.87 | 1.25 | 2.79 | 1.67 | 1.10 | 2.54 | 4.61 | 2.59 | 8.19 | 4.19 | 2.43 | 7.24 |
|  | Distant | 1.42 | 0.89 | 2.26 | 1.48 | 0.93 | 2.36 | 3.42 | 2.00 | 5.86 | 2.83 | 1.62 | 4.95 | 2.21 | 0.92 | 5.32 | 2.24 | 0.72 | 6.96 |
|  | Unknown | 1.64 | 0.76 | 3.57 | 1.11 | 0.45 | 2.74 | 0.64 | 0.15 | 2.76 | 0.72 | 0.18 | 2.89 | 3.32 | 0.72 | 15.38 | 1.80 | 0.31 | 10.34 |
| Surgery | |  |  |  |  |  |  |  |  |  |  |  |  |  |  |  |  |  |  |
|  | No | 1.00 |  |  | 1.00 |  |  | 1.00 |  |  | 1.00 |  |  | 1.00 |  |  | 1.00 |  |  |
|  | Yes | 0.40 | 0.27 | 0.60 | 0.39 | 0.26 | 0.59 | 0.39 | 0.23 | 0.65 | 0.47 | 0.27 | 0.81 | 0.11 | 0.05 | 0.28 | 0.24 | 0.07 | 0.77 |
|  | Unknown | 2.75 | 0.65 | 11.73 | 2.67 | 1.08 | 6.62 | 2.73 | 0.61 | 12.35 | 3.20 | 1.71 | 5.99 |  |  |  |  |  |  |
| Radiation | |  |  |  |  |  |  |  |  |  |  |  |  |  |  |  |  |  |  |
|  | No | 1.00 |  |  | 1.00 |  |  | 1.00 |  |  | 1.00 |  |  | 1.00 |  |  | 1.00 |  |  |
|  | Unknown | 1.40 | 0.98 | 2.00 | 1.31 | 0.90 | 1.90 | 1.52 | 1.03 | 2.25 | 1.52 | 1.04 | 2.23 | 1.02 | 0.60 | 1.72 | 0.95 | 0.56 | 1.62 |
| Category | |  |  |  |  |  |  |  |  |  |  |  |  |  |  |  |  |  |  |
|  | >2005 | 1.00 |  |  | 1.00 |  |  | 1.00 |  |  | 1.00 |  |  | 1.00 |  |  | 1.00 |  |  |
|  | 1995-2005 | 0.89 | 0.53 | 1.48 | 0.79 | 0.47 | 1.32 | 0.81 | 0.32 | 2.02 | 0.68 | 0.23 | 2.01 | 0.69 | 0.23 | 2.06 | 0.55 | 0.18 | 1.68 |
|  | <1995 | 0.96 | 0.51 | 1.78 | 0.85 | 0.46 | 1.58 | 0.97 | 0.37 | 2.51 | 0.97 | 0.33 | 2.87 | 0.80 | 0.26 | 2.46 | 0.60 | 0.19 | 1.89 |
| Age | | 1.01 | 0.99 | 1.02 | 1.00 | 0.99 | 1.02 | 1.00 | 0.99 | 1.02 | 1.00 | 0.98 | 1.02 | 1.01 | 0.98 | 1.04 | 1.00 | 0.97 | 1.03 |

LABC: breast duct carcinoma with prior primary lung adenocarcinoma; SBC: single breast cancer; H.R: risk of all-cause death; SHR: risk of cancer-specific death; NHW: Non-Hispanic White; NHB: Non-Hispanic Black; NHA: Non-Hispanic Asian or Pacific Islander; All variables were used in multivariate analysis.

Tab S7 summary of single lung adenocarcinoma and breast duct carcinoma

|  | | Breast duct carcinoma | Lung adenocarcinoma |
| --- | --- | --- | --- |
| No | | 705725 | 262486 |
| Race | |  |  |
|  | NHW | 506183 | 195551 |
|  | NHB | 71746 | 30063 |
|  | NHA | 54668 | 20895 |
|  | Hispanic | 66047 | 14643 |
|  | Others | 7081 | 1334 |
| Year of diagnosis | | |  |
|  | >2005 | 498495 | 133655 |
|  | 1994-2006 | 311055 | 77583 |
|  | <1995 | 188836 | 51248 |
| Age of diagnosis | | |  |
|  | Mean | 50 | 67 |
|  | IQR | 49-70 | 59-75 |
| Sex | |  |  |
|  | Men | 5143 | 136514 |
|  | Women | 700582 | 125972 |
| Insurance | | |  |
|  | Uninsured | 6283 | 3945 |
|  | Insured | 323492 | 117131 |
|  | Unknown | 375950 | 141410 |
| Marital status | | |  |
|  | Single | 282551 | 110008 |
|  | Married | 392738 | 142293 |
|  | Unknown | 30436 | 10185 |
| Site | | Breast | lung |
| Histologic | | Duct carcinoma | Adenocarcinoma |
| Seerstage | |  |  |
|  | Localized | 620228 | 79657 |
|  | Regional | 38335 | 134552 |
|  | Distant | 8973 | 7560 |
|  | Unknown | 38189 | 40717 |
| Radiation | |  |  |
|  | No | 0 | 47 |
|  | Yes | 328247 | 108357 |
|  | Unknown | 377478 | 154082 |
| Surgery | |  |  |
|  | No | 39928 | 193059 |
|  | Yes | 660805 | 64377 |
|  | Unknown | 4992 | 5050 |
| Major cause of cancer-related death | | | |
|  | Breast | 47.94% | 0.16% |
|  | lung | 0.94% | 83.17% |
| Major cause of noncancer-related death | | | |
|  | Heart disease | 17.11% | 3.99% |
|  | Other Cause of Death | 8.60% | 0.07% |
|  | COPD | 3.22% | 1.98% |

NHW: Non-Hispanic White; NHB: Non-Hispanic Black; NHA: Non-Hispanic Asian or Pacific Islander; COPD: chronic obstructive pulmonary disease.

Tab S8 Balance test between patients with BCLA and single breast cancer in matched cohort

| Variable | Mean | |  |  |
| --- | --- | --- | --- | --- |
|  | Treated | Control | bias | p |
| 2.surgery#4.seerstage | <0.01 | <0.01 | 2.4 | 0.16 |
| 3.race#2.surgery | <0.01 | <0.01 | 1.5 | 0.32 |
| 5.race#3.seerstage | <0.01 | <0.01 | 1.4 | 0.32 |
| 2.insurance#4.seerstage | <0.01 | <0.01 | 2.9 | 0.35 |
| 1.insurance#2.surgery | <0.01 | <0.01 | 1.6 | 0.44 |
| 2.insurance#2.surgery | <0.01 | <0.01 | 1.9 | 0.48 |
| 2.surgery#1.radiation | <0.01 | <0.01 | 1.8 | 0.53 |
| 5.race#2.category | <0.01 | <0.01 | 1.2 | 0.56 |
| 1.surgery | 0.96 | 0.96 | 1.2 | 0.57 |
| 1.insurance#3.seerstage | 0.01 | 0.01 | 1.6 | 0.59 |
| 5.race#c.age | 0.26 | 0.21 | 0.9 | 0.61 |
| 3.seerstage#c.age | 1.49 | 1.38 | 1.4 | 0.62 |
| 3.seerstage | 0.02 | 0.02 | 1.3 | 0.63 |
| 2.surgery#2.category | <0.01 | <0.01 | 1.2 | 0.66 |
| 4.race#4.seerstage | <0.01 | <0.01 | 0.5 | 0.66 |
| 1.surgery#c.age | 60.37 | 60.53 | 0.9 | 0.68 |
| 2.surgery#2.seerstage | <0.01 | <0.01 | 1 | 0.71 |
| 1.insurance#5.race | <0.01 | <0.01 | 0.6 | 0.71 |
| 5.race#3.category | <0.01 | <0.01 | 1 | 0.71 |
| 5.race#2.seerstage | <0.01 | <0.01 | 1 | 0.71 |
| 4.race#2.radiation | 0.01 | 0.01 | 0.5 | 0.71 |
| 5.race | <0.01 | <0.01 | 0.7 | 0.72 |
| 1.insurance#1.radiation | 0.22 | 0.22 | 0.8 | 0.73 |
| 3.race#4.seerstage | <0.01 | <0.01 | 0.5 | 0.74 |
| 4.race#3.seerstage | <0.01 | <0.01 | 0.8 | 0.74 |
| 3.race#2.radiation | 0.02 | 0.02 | 0.7 | 0.75 |
| 4.race#2.seerstage | 0.01 | 0.01 | 0.9 | 0.76 |
| 5.race#2.radiation | <0.01 | <0.01 | 0.5 | 0.76 |
| 2.insurance | 0.73 | 0.73 | 0.7 | 0.77 |
| 4.race#c.age | 2.93 | 2.84 | 0.6 | 0.77 |
| 4.race | 0.05 | 0.05 | 0.6 | 0.78 |
| 2.insurance#c.age | 44.64 | 44.83 | 0.6 | 0.78 |
| 2.surgery#2.radiation | <0.01 | <0.01 | 0.6 | 0.78 |
| 1.insurance#c.age | 18.17 | 17.97 | 0.7 | 0.78 |
| 2.surgery#c.age | 0.23 | 0.21 | 0.6 | 0.80 |
| 3.race#1.radiation | 0.04 | 0.04 | 0.6 | 0.81 |
| 1.insurance | 0.27 | 0.27 | 0.5 | 0.81 |
| 2.race#3.seerstage | <0.01 | <0.01 | 0.7 | 0.81 |
| 2.seerstage#c.age | 16.62 | 16.78 | 0.7 | 0.81 |
| 1.insurance#4.seerstage | 0.01 | 0.01 | 0.3 | 0.82 |
| 3.race#3.category | 0.01 | 0.01 | 0.6 | 0.82 |
| 2.race#1.surgery | 0.08 | 0.08 | 0.5 | 0.83 |
| 3.category#3.seerstage | <0.01 | <0.01 | 0.5 | 0.83 |
| 5.race#1.radiation | <0.01 | <0.01 | 0.5 | 0.83 |
| 1.surgery#2.seerstage | 0.27 | 0.27 | 0.7 | 0.83 |
| 2.seerstage | 0.27 | 0.28 | 0.7 | 0.83 |
| 4.seerstage | 0.01 | 0.01 | 0.3 | 0.84 |
| 2.surgery | <0.01 | <0.01 | 0.5 | 0.84 |
| 2.insurance#2.seerstage | 0.20 | 0.21 | 0.7 | 0.84 |
| 2.race#2.seerstage | 0.03 | 0.03 | 0.6 | 0.84 |
| 2.insurance#5.race | <0.01 | <0.01 | 0.4 | 0.84 |
| c.age#c.age | 4091.80 | 4085.30 | 0.4 | 0.85 |
| 2.insurance#1.radiation | 0.44 | 0.45 | 0.5 | 0.85 |
| 1.insurance#4.race | 0.02 | 0.02 | 0.3 | 0.85 |
| 2.insurance#1.surgery | 0.71 | 0.72 | 0.4 | 0.85 |
| 5.race#1.surgery | <0.01 | <0.01 | 0.4 | 0.86 |
| 4.race#1.surgery | 0.04 | 0.04 | 0.3 | 0.86 |
| 1.insurance#2.radiation | 0.05 | 0.05 | 0.3 | 0.86 |
| 4.race#2.category | 0.02 | 0.02 | 0.4 | 0.87 |
| 1.radiation#c.age | 42.65 | 42.53 | 0.4 | 0.87 |
| 1.surgery#1.radiation | 0.65 | 0.65 | 0.4 | 0.88 |
| age | 62.99 | 62.95 | 0.3 | 0.88 |
| 2.category#2.seerstage | 0.12 | 0.12 | 0.5 | 0.88 |
| 2.insurance#4.race | 0.03 | 0.03 | 0.3 | 0.89 |
| 4.race#3.category | 0.01 | 0.01 | 0.3 | 0.89 |
| 1.radiation | 0.67 | 0.67 | 0.3 | 0.90 |
| 2.radiation | 0.33 | 0.33 | 0.3 | 0.90 |
| 4.race#1.radiation | 0.04 | 0.04 | 0.3 | 0.90 |
| 2.insurance#3.seerstage | 0.01 | 0.01 | 0.3 | 0.90 |
| 2.radiation#c.age | 20.34 | 20.43 | 0.3 | 0.91 |
| 4.seerstage#c.age | 0.93 | 0.95 | 0.2 | 0.91 |
| 2.insurance#2.race | 0.06 | 0.06 | 0.3 | 0.92 |
| 1.surgery#3.seerstage | 0.01 | 0.01 | 0.3 | 0.92 |
| 1.surgery#2.category | 0.40 | 0.40 | 0.2 | 0.92 |
| 3.race#2.seerstage | 0.02 | 0.02 | 0.3 | 0.92 |
| 1.insurance#2.seerstage | 0.07 | 0.07 | 0.3 | 0.93 |
| 2.race#3.category | 0.02 | 0.02 | 0.2 | 0.93 |
| 2.race | 0.09 | 0.09 | 0.2 | 0.93 |
| 3.category#1.radiation | 0.10 | 0.10 | 0.2 | 0.94 |
| 2.insurance#2.radiation | 0.28 | 0.28 | 0.2 | 0.94 |
| 2.category#1.radiation | 0.31 | 0.31 | 0.2 | 0.94 |
| 1.surgery#2.radiation | 0.31 | 0.31 | 0.2 | 0.94 |
| 3.race#2.category | 0.02 | 0.02 | 0.2 | 0.94 |
| 2.category | 0.41 | 0.41 | 0.2 | 0.94 |
| 2.race#2.radiation | 0.03 | 0.03 | 0.1 | 0.94 |
| 2.category#c.age | 26.01 | 26.06 | 0.2 | 0.95 |
| 2.race#2.category | 0.03 | 0.03 | 0.2 | 0.95 |
| 1.insurance#1.surgery | 0.24 | 0.24 | 0.1 | 0.96 |
| 3.category#c.age | 16.09 | 16.13 | 0.1 | 0.96 |
| 2.race#1.radiation | 0.06 | 0.06 | 0.1 | 0.96 |
| 2.race#c.age | 5.15 | 5.17 | 0.1 | 0.96 |
| 3.race#1.surgery | 0.06 | 0.06 | 0.1 | 0.96 |
| 3.category#2.seerstage | 0.08 | 0.08 | 0.1 | 0.97 |
| 3.race#c.age | 3.85 | 3.87 | 0.1 | 0.97 |
| 3.category#2.radiation | 0.18 | 0.18 | 0.1 | 0.98 |
| 3.category | 0.28 | 0.28 | 0.1 | 0.98 |
| 1.surgery#3.category | 0.28 | 0.28 | 0.1 | 0.98 |
| 3.race | 0.06 | 0.06 | 0 | 1.00 |
| 2.category#3.seerstage | 0.01 | 0.01 | 0 | 1.00 |
| 1.surgery#4.seerstage | 0.01 | 0.01 | 0 | 1.00 |
| 2.category#2.radiation | 0.10 | 0.10 | 0 | 1.00 |
| 1.insurance#2.race | 0.03 | 0.03 | 0 | 1.00 |
| 1.insurance#3.race | 0.02 | 0.02 | 0 | 1.00 |
| 2.insurance#3.race | 0.04 | 0.04 | 0 | 1.00 |

BCLA: breast duct carcinoma with subsequent primary lung adenocarcinoma; bias: absolutely standardized difference; p: t test between treated and control group

Tab S9 Balance test between group sTPC with BCLA and single breast cancer in matched cohort

| Variable | Mean | |  |  |
| --- | --- | --- | --- | --- |
|  | Treated | Control | bias | p |
| age | 67.01 | 66.88 | 1.10 | 0.84 |
| 2.seerstage | 0.28 | 0.28 | 0.50 | 0.95 |
| 3.seerstage | 0.07 | 0.07 | 2.00 | 0.81 |
| 4.seerstage | 0.08 | 0.09 | 2.30 | 0.74 |
| 1.radiation | 0.68 | 0.68 | 0.80 | 0.89 |
| 2.radiation | 0.32 | 0.32 | 0.80 | 0.89 |
| 2.category | 0.25 | 0.25 | 0.40 | 0.94 |
| 3.category | 0.10 | 0.10 | 0.60 | 0.92 |
| 2.race | 0.09 | 0.10 | 1.30 | 0.83 |
| 3.race | 0.07 | 0.08 | 1.50 | 0.81 |
| 4.race | 0.05 | 0.05 | 0.80 | 0.88 |
| 5.race | <0.01 | <0.01 | <0.01 | 1.00 |
| 1.marital | 0.44 | 0.44 | 0.80 | 0.90 |
| 2.marital | 0.04 | 0.03 | 3.90 | 0.52 |
| 1.insurance | 0.59 | 0.59 | 0.80 | 0.90 |
| 2.insurance | 0.40 | 0.41 | 1.20 | 0.85 |
| 1.surgery | 0.80 | 0.80 | 1.20 | 0.88 |
| 2.surgery | 0.02 | 0.02 | <0.01 | 1.00 |
| 1.surgery#2.seerstage | 0.24 | 0.24 | 0.60 | 0.94 |
| 1.surgery#3.seerstage | 0.03 | 0.03 | 1.50 | 0.86 |
| 1.surgery#4.seerstage | 0.05 | 0.05 | 0.90 | 0.89 |
| 2.surgery#2.seerstage | <0.01 | <0.01 | <0.01 | 1.00 |
| 2.surgery#4.seerstage | <0.01 | 0.01 | 4.20 | 0.65 |
| 2.category#2.seerstage | 0.07 | 0.07 | <0.01 | 1.00 |
| 2.category#3.seerstage | 0.01 | 0.01 | 2.10 | 0.78 |
| 3.category#2.seerstage | 0.02 | 0.02 | <0.01 | 1.00 |
| 3.category#3.seerstage | 0.01 | 0.01 | <0.01 | 1.00 |
| c.age#c.age | 4597.30 | 4582.00 | 1.00 | 0.86 |
| 1.surgery#1.radiation | 0.55 | 0.56 | 0.80 | 0.90 |
| 1.surgery#2.radiation | 0.25 | 0.25 | <0.01 | 1.00 |
| 2.surgery#1.radiation | 0.01 | 0.01 | 2.80 | 0.76 |
| 2.surgery#2.radiation | 0.01 | <0.01 | 3.00 | 0.65 |
| 1.surgery#2.category | 0.22 | 0.22 | <0.01 | 1.00 |
| 1.surgery#3.category | 0.10 | 0.10 | 0.60 | 0.92 |
| 2.surgery#2.category | <0.01 | <0.01 | <0.01 | 1.00 |
| 2.category#1.radiation | 0.16 | 0.16 | <0.01 | 1.00 |
| 2.category#2.radiation | 0.10 | 0.10 | 0.60 | 0.92 |
| 3.category#1.radiation | 0.03 | 0.03 | 1.00 | 0.85 |
| 3.category#2.radiation | 0.08 | 0.08 | <0.01 | 1.00 |
| 1.insurance#2.seerstage | 0.17 | 0.17 | <0.01 | 1.00 |
| 1.insurance#3.seerstage | 0.05 | 0.04 | 2.50 | 0.76 |
| 1.insurance#4.seerstage | 0.07 | 0.07 | <0.01 | 1.00 |
| 2.insurance#2.seerstage | 0.10 | 0.10 | <0.01 | 1.00 |
| 2.insurance#3.seerstage | 0.02 | 0.02 | <0.01 | 1.00 |
| 2.insurance#4.seerstage | 0.01 | 0.02 | 7.90 | 0.40 |
| 2.category#c.age | 16.79 | 16.68 | 0.40 | 0.95 |
| 3.category#c.age | 6.48 | 6.59 | 0.50 | 0.93 |
| 2.seerstage#c.age | 18.11 | 17.94 | 0.70 | 0.93 |
| 3.seerstage#c.age | 4.43 | 4.07 | 2.90 | 0.72 |
| 4.seerstage#c.age | 5.90 | 6.42 | 3.10 | 0.68 |
| 1.marital#2.seerstage | 0.13 | 0.12 | 1.50 | 0.85 |
| 1.marital#3.seerstage | 0.03 | 0.03 | 3.10 | 0.70 |
| 1.marital#4.seerstage | 0.02 | 0.03 | 4.80 | 0.44 |
| 2.marital#2.seerstage | 0.01 | 0.01 | 2.30 | 0.76 |
| 2.marital#3.seerstage | <0.01 | <0.01 | <0.01 | 1.00 |
| 2.marital#4.seerstage | 0.01 | 0.01 | 5.00 | 0.48 |
| 1.surgery#2.race | 0.06 | 0.07 | 2.20 | 0.70 |
| 1.surgery#3.race | 0.06 | 0.06 | <0.01 | 1.00 |
| 1.surgery#4.race | 0.03 | 0.03 | <0.01 | 1.00 |
| 1.surgery#5.race | <0.01 | <0.01 | <0.01 | 1.00 |
| 2.race#2.seerstage | 0.03 | 0.04 | 2.70 | 0.74 |
| 2.race#3.seerstage | 0.01 | 0.01 | 2.40 | 0.76 |
| 2.race#4.seerstage | 0.01 | 0.01 | <0.01 | 1.00 |
| 3.race#2.seerstage | 0.03 | 0.03 | <0.01 | 1.00 |
| 3.race#3.seerstage | 0.01 | <0.01 | 3.50 | 0.65 |
| 3.race#4.seerstage | 0.01 | 0.01 | 7.90 | 0.32 |
| 4.race#2.seerstage | 0.01 | <0.01 | 4.90 | 0.41 |
| 4.race#3.seerstage | <0.01 | <0.01 | <0.01 | 1.00 |
| 4.race#4.seerstage | <0.01 | <0.01 | <0.01 | 1.00 |
| 1.surgery#c.age | 53.56 | 53.70 | 0.60 | 0.94 |
| 2.surgery#c.age | 1.08 | 1.08 | 0.10 | 0.99 |
| 1.surgery#1.marital | 0.38 | 0.38 | 0.40 | 0.95 |
| 1.surgery#2.marital | 0.02 | 0.02 | 1.20 | 0.83 |
| 2.surgery#1.marital | 0.01 | 0.01 | 2.60 | 0.76 |
| 2.surgery#2.marital | <0.01 | <0.01 | 5.90 | 0.32 |
| 1.marital#2.race | 0.02 | 0.03 | 3.50 | 0.54 |
| 1.marital#3.race | 0.04 | 0.04 | 0.90 | 0.87 |
| 1.marital#4.race | 0.02 | 0.02 | 2.00 | 0.67 |
| 2.marital#2.race | <0.01 | <0.01 | <0.01 | 1.00 |
| 2.marital#4.race | <0.01 | <0.01 | <0.01 | 1.00 |
| 1.insurance#2.race | 0.07 | 0.07 | 0.80 | 0.90 |
| 1.insurance#3.race | 0.05 | 0.05 | <0.01 | 1.00 |
| 1.insurance#4.race | 0.03 | 0.03 | 2.00 | 0.70 |
| 2.insurance#2.race | 0.02 | 0.03 | 1.00 | 0.84 |
| 2.insurance#3.race | 0.02 | 0.03 | 2.40 | 0.68 |
| 2.insurance#4.race | 0.02 | 0.02 | 1.20 | 0.82 |
| 2.insurance#5.race | <0.01 | <0.01 | <0.01 | 1.00 |
| 2.race#2.category | 0.02 | 0.02 | <0.01 | 1.00 |
| 2.race#3.category | <0.01 | 0.01 | 2.10 | 0.65 |
| 3.race#2.category | 0.01 | 0.01 | <0.01 | 1.00 |
| 3.race#3.category | <0.01 | <0.01 | <0.01 | 1.00 |
| 4.race#2.category | 0.01 | 0.01 | <0.01 | 1.00 |
| 4.race#3.category | <0.01 | <0.01 | <0.01 | 1.00 |
| 2.race#c.age | 5.87 | 6.09 | 1.20 | 0.86 |
| 3.race#c.age | 4.56 | 4.89 | 2.00 | 0.76 |
| 4.race#c.age | 3.25 | 3.07 | 1.10 | 0.84 |
| 5.race#c.age | 0.12 | 0.12 | <0.01 | 1.00 |
| 1.surgery#1.insurance | 0.45 | 0.45 | 0.40 | 0.95 |
| 1.surgery#2.insurance | 0.35 | 0.35 | 0.40 | 0.95 |
| 2.surgery#1.insurance | 0.01 | 0.01 | 2.50 | 0.76 |
| 2.surgery#2.insurance | 0.01 | <0.01 | 3.40 | 0.65 |

BCLA: breast duct carcinoma with subsequent primary lung adenocarcinoma; bias: absolutely standardized difference; p: t test between treated and control group

Tab S10 Balance test between group mTPC1 with BCLA and single breast cancer in matched cohort

| Variable | Mean | |  |  |
| --- | --- | --- | --- | --- |
|  | Treated | Control | bias | p |
| age | 65.92 | 65.91 | 0.10 | 0.98 |
| 2.seerstage | 0.24 | 0.25 | 0.30 | 0.96 |
| 3.seerstage | 0.03 | 0.03 | 0.70 | 0.89 |
| 1.radiation | 0.71 | 0.71 | 0.80 | 0.85 |
| 2.radiation | 0.29 | 0.29 | 0.80 | 0.85 |
| 1.surgery | 0.98 | 0.98 | 1.20 | 0.74 |
| 2.surgery | <0.01 | <0.01 | 2.00 | 0.56 |
| 2.race | 0.08 | 0.08 | 1.30 | 0.75 |
| 3.race | 0.06 | 0.06 | 0.80 | 0.86 |
| 4.race | 0.05 | 0.05 | 0.40 | 0.92 |
| 5.race | <0.01 | <0.01 | <0.01 | 1.00 |
| 2.category | 0.36 | 0.36 | 0.40 | 0.93 |
| 3.category | 0.16 | 0.17 | 0.50 | 0.91 |
| 2.sex | 0.99 | 0.99 | 1.90 | 0.69 |
| c.age#c.age | 4466.00 | 4462.70 | 0.20 | 0.96 |
| 2.category#1.radiation | 0.26 | 0.26 | 0.50 | 0.92 |
| 2.category#2.radiation | 0.10 | 0.10 | 1.10 | 0.77 |
| 3.category#1.radiation | 0.04 | 0.05 | 0.90 | 0.84 |
| 3.category#2.radiation | 0.12 | 0.12 | <0.01 | 1.00 |
| 1.surgery#2.seerstage | 0.24 | 0.24 | <0.01 | 1.00 |
| 1.surgery#3.seerstage | 0.02 | 0.02 | 0.80 | 0.88 |
| 2.category#2.seerstage | 0.10 | 0.10 | 1.30 | 0.83 |
| 2.category#3.seerstage | 0.01 | 0.01 | <0.01 | 1.00 |
| 3.category#2.seerstage | 0.04 | 0.04 | 0.60 | 0.91 |
| 3.category#3.seerstage | <0.01 | <0.01 | 2.00 | 0.66 |
| 2.category#c.age | 23.75 | 23.66 | 0.30 | 0.95 |
| 3.category#c.age | 10.50 | 10.63 | 0.50 | 0.90 |
| 2.category#2.race | 0.02 | 0.02 | 1.80 | 0.66 |
| 2.category#3.race | 0.02 | 0.02 | <0.01 | 1.00 |
| 2.category#4.race | 0.02 | 0.02 | 0.60 | 0.87 |
| 2.category#5.race | <0.01 | <0.01 | 2.20 | 0.32 |
| 3.category#2.race | 0.01 | 0.01 | <0.01 | 1.00 |
| 3.category#4.race | <0.01 | <0.01 | <0.01 | 1.00 |
| 2.race#2.seerstage | 0.04 | 0.03 | 2.10 | 0.72 |
| 2.race#3.seerstage | <0.01 | <0.01 | 3.00 | 0.32 |
| 3.race#2.seerstage | 0.02 | 0.02 | <0.01 | 1.00 |
| 3.race#3.seerstage | <0.01 | <0.01 | <0.01 | 1.00 |
| 4.race#2.seerstage | 0.01 | 0.02 | 1.00 | 0.86 |
| 4.race#3.seerstage | <0.01 | <0.01 | 2.20 | 0.71 |
| 5.race#2.seerstage | <0.01 | <0.01 | 2.90 | 0.66 |
| 5.race#3.seerstage | <0.01 | <0.01 | 3.70 | 0.32 |
| 2.category#1.surgery | 0.36 | 0.35 | 0.60 | 0.89 |
| 3.category#1.surgery | 0.16 | 0.17 | 0.50 | 0.91 |
| 2.race#c.age | 4.96 | 4.69 | 1.60 | 0.71 |
| 3.race#c.age | 4.08 | 4.00 | 0.50 | 0.91 |
| 4.race#c.age | 3.28 | 3.35 | 0.50 | 0.91 |
| 5.race#c.age | 0.29 | 0.26 | 0.60 | 0.87 |
| 2.sex#c.age | 64.93 | 65.08 | 1.00 | 0.80 |
| 1.radiation#c.age | 46.68 | 46.92 | 0.80 | 0.86 |
| 2.radiation#c.age | 19.24 | 18.99 | 0.80 | 0.86 |
| 2.seerstage#c.age | 15.55 | 15.58 | 0.10 | 0.98 |
| 3.seerstage#c.age | 1.83 | 1.90 | 0.80 | 0.89 |
| 1.radiation#2.seerstage | 0.17 | 0.18 | 1.70 | 0.77 |
| 1.radiation#3.seerstage | 0.02 | 0.02 | 1.00 | 0.87 |
| 2.radiation#2.seerstage | 0.07 | 0.07 | 1.80 | 0.73 |
| 2.radiation#3.seerstage | 0.01 | 0.01 | <0.01 | 1.00 |

BCLA: breast duct carcinoma with subsequent primary lung adenocarcinoma; bias: absolutely standardized difference; p: t test between treated and control group

Tab S11 Balance test between group mTPC2 with BCLA and single breast cancer in matched cohort

| Variable | Mean | |  |  |
| --- | --- | --- | --- | --- |
|  | Treated | Control | bias | p |
| age | 60.31 | 60.30 | <0.01 | 1.00 |
| 2.category | 0.48 | 0.48 | 0.20 | 0.95 |
| 3.category | 0.38 | 0.39 | 0.30 | 0.92 |
| 2.seerstage | 0.29 | 0.29 | 0.80 | 0.86 |
| 3.seerstage | 0.01 | 0.01 | 1.80 | 0.59 |
| 1.surgery | 0.99 | 1.00 | 2.10 | 0.39 |
| 2.surgery | <0.01 | <0.01 | 3.90 | 0.16 |
| 2.radiation | 0.35 | 0.35 | 0.40 | 0.89 |
| 1.insurance | 0.10 | 0.10 | 0.50 | 0.83 |
| 2.insurance | 0.90 | 0.90 | 0.50 | 0.83 |
| 2.race | 0.09 | 0.08 | 2.40 | 0.45 |
| 3.race | 0.06 | 0.06 | <0.01 | 1.00 |
| 4.race | 0.04 | 0.04 | 1.50 | 0.58 |
| 5.race | 0.01 | <0.01 | 1.90 | 0.49 |
| 1.marital | 0.58 | 0.58 | 0.10 | 0.97 |
| 2.marital | 0.03 | 0.03 | <0.01 | 1.00 |
| 2.sex | 1.00 | 1.00 | 2.50 | 0.44 |
| c.age#c.age | 3751.30 | 3750.50 | 0.10 | 0.98 |
| 2.category#c.age | 29.77 | 29.74 | 0.10 | 0.98 |
| 3.category#c.age | 21.77 | 21.94 | 0.60 | 0.85 |
| 2.seerstage#2.category | 0.14 | 0.14 | 0.20 | 0.96 |
| 2.seerstage#3.category | 0.12 | 0.12 | 0.70 | 0.88 |
| 3.seerstage#2.category | <0.01 | <0.01 | 0.90 | 0.78 |
| 3.seerstage#3.category | <0.01 | <0.01 | <0.01 | 1.00 |
| 2.radiation#2.category | 0.10 | 0.10 | 1.00 | 0.71 |
| 2.radiation#3.category | 0.23 | 0.24 | 0.10 | 0.97 |
| 1.surgery#2.seerstage | 0.29 | 0.29 | 1.00 | 0.83 |
| 1.surgery#3.seerstage | 0.01 | 0.01 | 0.70 | 0.85 |
| 2.surgery#2.seerstage | <0.01 | <0.01 | 3.10 | 0.32 |
| 2.seerstage#c.age | 16.83 | 16.96 | 0.70 | 0.88 |
| 3.seerstage#c.age | 0.51 | 0.41 | 1.90 | 0.54 |
| 1.surgery#2.category | 0.48 | 0.48 | 0.20 | 0.95 |
| 1.surgery#3.category | 0.38 | 0.39 | 0.30 | 0.92 |
| 1.insurance#2.seerstage | 0.03 | 0.03 | 0.80 | 0.85 |
| 1.insurance#3.seerstage | <0.01 | <0.01 | 4.90 | 0.10 |
| 2.insurance#2.seerstage | 0.26 | 0.26 | 0.50 | 0.91 |
| 2.insurance#3.seerstage | 0.01 | 0.01 | 0.70 | 0.84 |
| 2.race#2.radiation | 0.03 | 0.03 | 0.50 | 0.85 |
| 3.race#2.radiation | 0.03 | 0.03 | 0.90 | 0.77 |
| 4.race#2.radiation | 0.01 | 0.01 | 1.00 | 0.58 |
| 5.race#2.radiation | <0.01 | <0.01 | <0.01 | 1.00 |
| 2.race#2.seerstage | 0.03 | 0.02 | 5.30 | 0.21 |
| 2.race#3.seerstage | <0.01 | <0.01 | 1.70 | 0.56 |
| 3.race#2.seerstage | 0.01 | 0.01 | <0.01 | 1.00 |
| 3.race#3.seerstage | <0.01 | <0.01 | 2.60 | 0.32 |
| 4.race#2.seerstage | 0.02 | 0.01 | 3.30 | 0.43 |
| 5.race#2.seerstage | <0.01 | <0.01 | 2.20 | 0.56 |
| 2.race#c.age | 5.06 | 4.63 | 2.60 | 0.42 |
| 3.race#c.age | 3.51 | 3.54 | 0.20 | 0.94 |
| 4.race#c.age | 2.66 | 2.44 | 1.60 | 0.58 |
| 5.race#c.age | 0.28 | 0.20 | 1.70 | 0.49 |
| 2.race#1.surgery | 0.08 | 0.08 | 1.90 | 0.56 |
| 2.race#2.surgery | <0.01 | <0.01 | 2.90 | 0.32 |
| 3.race#1.surgery | 0.06 | 0.06 | <0.01 | 1.00 |
| 4.race#1.surgery | 0.04 | 0.04 | 1.50 | 0.57 |
| 5.race#1.surgery | 0.01 | <0.01 | 1.90 | 0.49 |
| 1.marital#2.race | 0.03 | 0.03 | 1.80 | 0.57 |
| 1.marital#3.race | 0.04 | 0.04 | 0.30 | 0.93 |
| 1.marital#4.race | 0.02 | 0.02 | 1.50 | 0.55 |
| 1.marital#5.race | <0.01 | <0.01 | 1.80 | 0.48 |
| 2.marital#2.race | 0.01 | 0.01 | <0.01 | 1.00 |
| 2.marital#3.race | <0.01 | <0.01 | 2.90 | 0.16 |
| 2.marital#4.race | <0.01 | <0.01 | 2.40 | 0.16 |
| 2.marital#5.race | <0.01 | <0.01 | 1.60 | 0.66 |
| 2.race#2.category | 0.04 | 0.04 | 1.60 | 0.63 |
| 2.race#3.category | 0.03 | 0.03 | 1.10 | 0.77 |
| 3.race#2.category | 0.03 | 0.03 | 0.30 | 0.93 |
| 3.race#3.category | 0.02 | 0.02 | 0.40 | 0.91 |
| 4.race#2.category | 0.03 | 0.03 | <0.01 | 1.00 |
| 4.race#3.category | 0.01 | 0.01 | 3.10 | 0.34 |
| 5.race#2.category | <0.01 | <0.01 | 0.90 | 0.76 |
| 5.race#3.category | <0.01 | <0.01 | 1.40 | 0.66 |
| 2.radiation#1.surgery | 0.35 | 0.35 | 0.60 | 0.84 |
| 2.radiation#2.surgery | <0.01 | <0.01 | 3.90 | 0.16 |
| 2.race#1.insurance | 0.01 | 0.01 | 1.90 | 0.38 |
| 2.race#2.insurance | 0.08 | 0.07 | 1.70 | 0.62 |
| 3.race#1.insurance | 0.01 | 0.01 | 0.40 | 0.85 |
| 3.race#2.insurance | 0.05 | 0.05 | 0.20 | 0.94 |
| 4.race#1.insurance | 0.01 | 0.01 | 0.40 | 0.84 |
| 4.race#2.insurance | 0.04 | 0.04 | 1.50 | 0.61 |
| 5.race#1.insurance | <0.01 | <0.01 | <0.01 | 1.00 |
| 5.race#2.insurance | 0.01 | <0.01 | 2.20 | 0.47 |
| 1.marital#2.category | 0.26 | 0.26 | 0.40 | 0.91 |
| 1.marital#3.category | 0.25 | 0.25 | 0.10 | 0.97 |
| 2.marital#2.category | 0.02 | 0.02 | <0.01 | 1.00 |
| 2.marital#3.category | 0.01 | 0.01 | 1.90 | 0.61 |
| 2.radiation#c.age | 20.51 | 20.65 | 0.40 | 0.89 |
| 1.marital#2.seerstage | 0.17 | 0.16 | 0.40 | 0.93 |
| 1.marital#3.seerstage | <0.01 | <0.01 | 0.80 | 0.81 |
| 2.marital#2.seerstage | 0.01 | 0.01 | 1.90 | 0.68 |
| 2.marital#3.seerstage | <0.01 | <0.01 | <0.01 | 1.00 |
| 1.insurance#c.age | 6.66 | 6.54 | 0.50 | 0.85 |
| 2.insurance#c.age | 53.62 | 53.74 | 0.50 | 0.86 |
| 1.marital#2.radiation | 0.22 | 0.22 | 0.20 | 0.94 |
| 2.marital#2.radiation | 0.01 | 0.01 | 0.90 | 0.73 |
| 2.sex#2.category | 0.48 | 0.48 | 0.40 | 0.90 |
| 2.sex#3.category | 0.38 | 0.38 | 0.50 | 0.89 |
| 2.sex#2.race | 0.09 | 0.08 | 2.10 | 0.52 |
| 2.sex#3.race | 0.06 | 0.06 | <0.01 | 1.00 |
| 2.sex#4.race | 0.04 | 0.04 | 1.50 | 0.58 |
| 2.sex#5.race | 0.01 | <0.01 | 1.90 | 0.49 |
| 1.marital#1.surgery | 0.58 | 0.58 | 0.10 | 0.97 |
| 2.marital#1.surgery | 0.03 | 0.03 | 0.60 | 0.84 |
| 1.marital#1.insurance | 0.06 | 0.06 | <0.01 | 1.00 |
| 1.marital#2.insurance | 0.52 | 0.52 | 0.10 | 0.97 |
| 2.marital#1.insurance | <0.01 | <0.01 | 1.90 | 0.18 |
| 2.marital#2.insurance | 0.02 | 0.03 | 1.00 | 0.76 |

BCLA: breast duct carcinoma with subsequent primary lung adenocarcinoma; bias: absolutely standardized difference; p: t test between treated and control group

Tab S12 Balance test between patients with BCLA and single lung cancer in matched cohort

| Variable | Mean | |  |  |
| --- | --- | --- | --- | --- |
|  | Treated | Control | bias | p |
| age | 62.99 | 62.95 | 0.30 | 0.88 |
| 2.seerstage | 0.27 | 0.28 | 0.70 | 0.83 |
| 3.seerstage | 0.02 | 0.02 | 1.30 | 0.63 |
| 4.seerstage | 0.01 | 0.01 | 0.30 | 0.84 |
| 1.radiation | 0.67 | 0.67 | 0.30 | 0.90 |
| 2.radiation | 0.33 | 0.33 | 0.30 | 0.90 |
| 2.category | 0.41 | 0.41 | 0.20 | 0.94 |
| 3.category | 0.28 | 0.28 | 0.10 | 0.98 |
| 1.surgery | 0.96 | 0.96 | 1.20 | 0.57 |
| 2.surgery | <0.01 | <0.01 | 0.50 | 0.84 |
| 2.race | 0.09 | 0.09 | 0.20 | 0.93 |
| 3.race | 0.06 | 0.06 | <0.01 | 1.00 |
| 4.race | 0.05 | 0.05 | 0.60 | 0.78 |
| 5.race | <0.01 | <0.01 | 0.70 | 0.72 |
| 1.insurance | 0.27 | 0.27 | 0.50 | 0.81 |
| 2.insurance | 0.73 | 0.73 | 0.70 | 0.77 |
| 2.category#2.seerstage | 0.12 | 0.12 | 0.50 | 0.88 |
| 2.category#3.seerstage | 0.01 | 0.01 | <0.01 | 1.00 |
| 3.category#2.seerstage | 0.08 | 0.08 | 0.10 | 0.97 |
| 3.category#3.seerstage | <0.01 | <0.01 | 0.50 | 0.83 |
| c.age#c.age | 4091.80 | 4085.30 | 0.40 | 0.85 |
| 2.category#c.age | 26.01 | 26.06 | 0.20 | 0.95 |
| 3.category#c.age | 16.09 | 16.13 | 0.10 | 0.96 |
| 1.surgery#2.seerstage | 0.27 | 0.27 | 0.70 | 0.83 |
| 1.surgery#3.seerstage | 0.01 | 0.01 | 0.30 | 0.92 |
| 1.surgery#4.seerstage | 0.01 | 0.01 | <0.01 | 1.00 |
| 2.surgery#2.seerstage | <0.01 | <0.01 | 1.00 | 0.71 |
| 2.surgery#4.seerstage | <0.01 | <0.01 | 2.40 | 0.16 |
| 2.category#1.radiation | 0.31 | 0.31 | 0.20 | 0.94 |
| 2.category#2.radiation | 0.10 | 0.10 | <0.01 | 1.00 |
| 3.category#1.radiation | 0.10 | 0.10 | 0.20 | 0.94 |
| 3.category#2.radiation | 0.18 | 0.18 | 0.10 | 0.98 |
| 1.surgery#2.category | 0.40 | 0.40 | 0.20 | 0.92 |
| 1.surgery#3.category | 0.28 | 0.28 | 0.10 | 0.98 |
| 2.surgery#2.category | <0.01 | <0.01 | 1.20 | 0.66 |
| 2.seerstage#c.age | 16.62 | 16.78 | 0.70 | 0.81 |
| 3.seerstage#c.age | 1.49 | 1.38 | 1.40 | 0.62 |
| 4.seerstage#c.age | 0.93 | 0.95 | 0.20 | 0.91 |
| 1.insurance#2.seerstage | 0.07 | 0.07 | 0.30 | 0.93 |
| 1.insurance#3.seerstage | 0.01 | 0.01 | 1.60 | 0.59 |
| 1.insurance#4.seerstage | 0.01 | 0.01 | 0.30 | 0.82 |
| 2.insurance#2.seerstage | 0.20 | 0.21 | 0.70 | 0.84 |
| 2.insurance#3.seerstage | 0.01 | 0.01 | 0.30 | 0.90 |
| 2.insurance#4.seerstage | <0.01 | <0.01 | 2.90 | 0.35 |
| 1.surgery#1.radiation | 0.65 | 0.65 | 0.40 | 0.88 |
| 1.surgery#2.radiation | 0.31 | 0.31 | 0.20 | 0.94 |
| 2.surgery#1.radiation | <0.01 | <0.01 | 1.80 | 0.53 |
| 2.surgery#2.radiation | <0.01 | <0.01 | 0.60 | 0.78 |
| 1.insurance#c.age | 18.17 | 17.97 | 0.70 | 0.78 |
| 2.insurance#c.age | 44.64 | 44.83 | 0.60 | 0.78 |
| 2.race#c.age | 5.15 | 5.17 | 0.10 | 0.96 |
| 3.race#c.age | 3.85 | 3.87 | 0.10 | 0.97 |
| 4.race#c.age | 2.93 | 2.84 | 0.60 | 0.77 |
| 5.race#c.age | 0.26 | 0.21 | 0.90 | 0.61 |
| 1.insurance#2.race | 0.03 | 0.03 | <0.01 | 1.00 |
| 1.insurance#3.race | 0.02 | 0.02 | <0.01 | 1.00 |
| 1.insurance#4.race | 0.02 | 0.02 | 0.30 | 0.85 |
| 1.insurance#5.race | <0.01 | <0.01 | 0.60 | 0.71 |
| 2.insurance#2.race | 0.06 | 0.06 | 0.30 | 0.92 |
| 2.insurance#3.race | 0.04 | 0.04 | <0.01 | 1.00 |
| 2.insurance#4.race | 0.03 | 0.03 | 0.30 | 0.89 |
| 2.insurance#5.race | <0.01 | <0.01 | 0.40 | 0.84 |
| 2.race#2.category | 0.03 | 0.03 | 0.20 | 0.95 |
| 2.race#3.category | 0.02 | 0.02 | 0.20 | 0.93 |
| 3.race#2.category | 0.02 | 0.02 | 0.20 | 0.94 |
| 3.race#3.category | 0.01 | 0.01 | 0.60 | 0.82 |
| 4.race#2.category | 0.02 | 0.02 | 0.40 | 0.87 |
| 4.race#3.category | 0.01 | 0.01 | 0.30 | 0.89 |
| 5.race#2.category | <0.01 | <0.01 | 1.20 | 0.56 |
| 5.race#3.category | <0.01 | <0.01 | 1.00 | 0.71 |
| 2.race#1.radiation | 0.06 | 0.06 | 0.10 | 0.96 |
| 2.race#2.radiation | 0.03 | 0.03 | 0.10 | 0.94 |
| 3.race#1.radiation | 0.04 | 0.04 | 0.60 | 0.81 |
| 3.race#2.radiation | 0.02 | 0.02 | 0.70 | 0.75 |
| 4.race#1.radiation | 0.04 | 0.04 | 0.30 | 0.90 |
| 4.race#2.radiation | 0.01 | 0.01 | 0.50 | 0.71 |
| 5.race#1.radiation | <0.01 | <0.01 | 0.50 | 0.83 |
| 5.race#2.radiation | <0.01 | <0.01 | 0.50 | 0.76 |
| 2.race#1.surgery | 0.08 | 0.08 | 0.50 | 0.83 |
| 2.race#2.surgery | <0.01 | <0.01 | <0.01 | 1.00 |
| 3.race#1.surgery | 0.06 | 0.06 | 0.10 | 0.96 |
| 3.race#2.surgery | <0.01 | <0.01 | 1.50 | 0.32 |
| 4.race#1.surgery | 0.04 | 0.04 | 0.30 | 0.86 |
| 5.race#1.surgery | <0.01 | <0.01 | 0.40 | 0.86 |
| 2.race#2.seerstage | 0.03 | 0.03 | 0.60 | 0.84 |
| 2.race#3.seerstage | <0.01 | <0.01 | 0.70 | 0.81 |
| 2.race#4.seerstage | <0.01 | <0.01 | <0.01 | 1.00 |
| 3.race#2.seerstage | 0.02 | 0.02 | 0.30 | 0.92 |
| 3.race#3.seerstage | <0.01 | <0.01 | <0.01 | 1.00 |
| 3.race#4.seerstage | <0.01 | <0.01 | 0.50 | 0.74 |
| 4.race#2.seerstage | 0.01 | 0.01 | 0.90 | 0.76 |
| 4.race#3.seerstage | <0.01 | <0.01 | 0.80 | 0.74 |
| 4.race#4.seerstage | <0.01 | <0.01 | 0.50 | 0.66 |
| 5.race#2.seerstage | <0.01 | <0.01 | 1.00 | 0.71 |
| 5.race#3.seerstage | <0.01 | <0.01 | 1.40 | 0.32 |
| 1.surgery#c.age | 60.37 | 60.53 | 0.90 | 0.68 |
| 2.surgery#c.age | 0.23 | 0.21 | 0.60 | 0.80 |
| 1.insurance#1.radiation | 0.22 | 0.22 | 0.80 | 0.73 |
| 1.insurance#2.radiation | 0.05 | 0.05 | 0.30 | 0.86 |
| 2.insurance#1.radiation | 0.44 | 0.45 | 0.50 | 0.85 |
| 2.insurance#2.radiation | 0.28 | 0.28 | 0.20 | 0.94 |
| 1.radiation#c.age | 42.65 | 42.53 | 0.40 | 0.87 |
| 2.radiation#c.age | 20.34 | 20.43 | 0.30 | 0.91 |
| 1.insurance#1.surgery | 0.24 | 0.24 | 0.10 | 0.96 |
| 1.insurance#2.surgery | <0.01 | <0.01 | 1.60 | 0.44 |
| 2.insurance#1.surgery | 0.71 | 0.72 | 0.40 | 0.85 |
| 2.insurance#2.surgery | <0.01 | <0.01 | 1.90 | 0.48 |

BCLA: breast duct carcinoma with subsequent primary lung adenocarcinoma; bias: absolutely standardized difference; p: t test between treated and control group

Tab S13 Balance test between group sTPC with BCLA and single lung cancer in matched cohort

| Variable | Mean | |  |  |
| --- | --- | --- | --- | --- |
|  | Treated | Control | bias | p |
| age | 68.41 | 68.29 | 1.00 | 0.91 |
| 1.surgery | 0.58 | 0.59 | 1.20 | 0.92 |
| 2.surgery | 0.01 | <0.01 | 6.40 | 0.56 |
| 1.radiation | 0.37 | 0.37 | 1.00 | 0.92 |
| 2.radiation | 0.63 | 0.63 | 1.00 | 0.92 |
| 2.seerstage | 0.12 | 0.11 | 1.70 | 0.88 |
| 3.seerstage | 0.10 | 0.11 | 6.50 | 0.64 |
| 4.seerstage | 0.08 | 0.08 | <0.01 | 1.00 |
| 2.race | 0.11 | 0.11 | 3.10 | 0.76 |
| 3.race | 0.06 | 0.06 | 1.90 | 0.84 |
| 4.race | 0.04 | 0.04 | 2.00 | 0.81 |
| 1.insurance | 0.55 | 0.54 | 1.00 | 0.92 |
| 2.insurance | 0.44 | 0.46 | 2.90 | 0.77 |
| 1.marital | 0.41 | 0.39 | 2.90 | 0.77 |
| 2.marital | 0.07 | 0.06 | 4.40 | 0.69 |
| 2.seerstage#1.surgery | 0.10 | 0.09 | 2.00 | 0.87 |
| 3.seerstage#1.surgery | 0.03 | 0.04 | 7.50 | 0.59 |
| 4.seerstage#1.surgery | 0.02 | 0.02 | <0.01 | 1.00 |
| c.age#c.age | 4795.00 | 4777.60 | 1.10 | 0.90 |
| 1.insurance#2.seerstage | 0.06 | 0.06 | <0.01 | 1.00 |
| 1.insurance#3.seerstage | 0.05 | 0.05 | <0.01 | 1.00 |
| 1.insurance#4.seerstage | 0.06 | 0.06 | <0.01 | 1.00 |
| 2.insurance#2.seerstage | 0.05 | 0.05 | <0.01 | 1.00 |
| 2.insurance#3.seerstage | 0.05 | 0.06 | 8.90 | 0.52 |
| 2.insurance#4.seerstage | 0.02 | 0.02 | 4.80 | 0.74 |
| 2.race#c.age | 6.37 | 6.83 | 2.50 | 0.81 |
| 3.race#c.age | 4.18 | 4.53 | 2.20 | 0.84 |
| 4.race#c.age | 2.91 | 2.58 | 2.20 | 0.80 |
| 2.race#2.seerstage | 0.01 | 0.01 | 4.50 | 0.65 |
| 2.race#3.seerstage | 0.02 | 0.04 | 23.90 | 0.16 |
| 2.race#4.seerstage | 0.01 | 0.01 | <0.01 | 1.00 |
| 3.race#2.seerstage | 0.01 | 0.01 | <0.01 | 1.00 |
| 3.race#4.seerstage | <0.01 | <0.01 | <0.01 | 1.00 |
| 4.race#2.seerstage | <0.01 | <0.01 | <0.01 | 1.00 |
| 4.race#3.seerstage | 0.01 | <0.01 | 6.70 | 0.56 |
| 1.marital#2.seerstage | 0.06 | 0.06 | <0.01 | 1.00 |
| 1.marital#3.seerstage | 0.03 | 0.02 | 7.10 | 0.56 |
| 1.marital#4.seerstage | 0.01 | 0.01 | <0.01 | 1.00 |
| 2.marital#2.seerstage | <0.01 | <0.01 | <0.01 | 1.00 |
| 2.marital#4.seerstage | 0.01 | 0.01 | <0.01 | 1.00 |
| 1.insurance#2.race | 0.04 | 0.05 | 4.60 | 0.63 |
| 1.insurance#3.race | 0.05 | 0.05 | 2.30 | 0.82 |
| 1.insurance#4.race | 0.02 | 0.02 | 2.60 | 0.74 |
| 2.insurance#2.race | 0.06 | 0.07 | 4.20 | 0.69 |
| 2.insurance#3.race | 0.01 | 0.01 | <0.01 | 1.00 |
| 2.insurance#4.race | 0.02 | 0.02 | <0.01 | 1.00 |
| 2.seerstage#c.age | 8.17 | 7.93 | 1.30 | 0.91 |
| 3.seerstage#c.age | 6.35 | 7.15 | 5.60 | 0.68 |
| 4.seerstage#c.age | 5.74 | 5.92 | 1.00 | 0.93 |
| 1.marital#2.race | 0.03 | 0.03 | 2.60 | 0.78 |
| 1.marital#3.race | 0.04 | 0.04 | <0.01 | 1.00 |
| 1.marital#4.race | 0.02 | 0.01 | 2.70 | 0.70 |
| 2.marital#2.race | 0.01 | <0.01 | 5.70 | 0.56 |
| 2.race#1.surgery | 0.03 | 0.04 | 2.00 | 0.79 |
| 2.race#2.surgery | <0.01 | <0.01 | 9.50 | 0.32 |
| 3.race#1.surgery | 0.03 | 0.03 | <0.01 | 1.00 |
| 4.race#1.surgery | 0.02 | 0.02 | <0.01 | 1.00 |
| 2.seerstage#1.radiation | 0.06 | 0.06 | <0.01 | 1.00 |
| 2.seerstage#2.radiation | 0.06 | 0.05 | 2.30 | 0.83 |
| 3.seerstage#1.radiation | 0.04 | 0.05 | 10.40 | 0.48 |
| 3.seerstage#2.radiation | 0.06 | 0.06 | <0.01 | 1.00 |
| 4.seerstage#1.radiation | 0.02 | 0.02 | 3.10 | 0.74 |
| 4.seerstage#2.radiation | 0.06 | 0.06 | 2.40 | 0.84 |
| 1.surgery#c.age | 40.15 | 40.42 | 1.00 | 0.94 |
| 2.surgery#c.age | 0.67 | 0.31 | 7.00 | 0.53 |
| 1.insurance#c.age | 38.25 | 37.89 | 1.10 | 0.92 |
| 2.insurance#c.age | 29.64 | 30.41 | 2.30 | 0.82 |
| 1.insurance#1.surgery | 0.26 | 0.27 | 1.00 | 0.91 |
| 1.insurance#2.surgery | <0.01 | <0.01 | <0.01 | 1.00 |
| 2.insurance#1.surgery | 0.32 | 0.32 | 1.00 | 0.92 |
| 2.insurance#2.surgery | <0.01 | <0.01 | 9.20 | 0.32 |
| 1.marital#1.surgery | 0.28 | 0.28 | <0.01 | 1.00 |
| 1.marital#2.surgery | <0.01 | <0.01 | <0.01 | 1.00 |
| 2.marital#1.surgery | 0.02 | 0.02 | <0.01 | 1.00 |
| 2.marital#2.surgery | <0.01 | <0.01 | 9.50 | 0.32 |

BCLA: breast duct carcinoma with subsequent primary lung adenocarcinoma; bias: absolutely standardized difference; p: t test between treated and control group

Tab S14 Balance test between group mTPC1 with BCLA and single lung cancer in matched cohort

| Variable | Mean | |  |  |
| --- | --- | --- | --- | --- |
|  | Treated | Control | bias | p |
| age | 68.65 | 68.53 | 1.00 | 0.81 |
| 2.sex | 0.99 | 0.99 | 0.30 | 0.85 |
| 2.seerstage | 0.24 | 0.26 | 3.40 | 0.41 |
| 3.seerstage | 0.36 | 0.36 | 1.40 | 0.82 |
| 4.seerstage | 0.12 | 0.11 | 3.40 | 0.49 |
| 1.surgery | 0.47 | 0.48 | 1.10 | 0.82 |
| 2.surgery | 0.01 | 0.01 | 1.40 | 0.80 |
| 2.category | 0.28 | 0.28 | <0.01 | 1.00 |
| 3.category | 0.12 | 0.12 | <0.01 | 1.00 |
| 1.insurance | 0.55 | 0.56 | 2.00 | 0.66 |
| 2.insurance | 0.45 | 0.44 | 1.80 | 0.69 |
| 1.marital | 0.47 | 0.48 | 1.80 | 0.69 |
| 2.marital | 0.05 | 0.03 | 7.80 | 0.07 |
| 2.race | 0.08 | 0.08 | 0.30 | 0.94 |
| 3.race | 0.06 | 0.06 | 0.40 | 0.93 |
| 4.race | 0.05 | 0.05 | 2.00 | 0.68 |
| 5.race | <0.01 | <0.01 | 3.40 | 0.48 |
| 1.radiation | 0.31 | 0.30 | 1.50 | 0.74 |
| 2.radiation | 0.69 | 0.70 | 1.50 | 0.74 |
| 2.category#2.seerstage | 0.09 | 0.09 | 2.00 | 0.59 |
| 2.category#3.seerstage | 0.12 | 0.12 | 2.00 | 0.73 |
| 3.category#2.seerstage | 0.01 | 0.02 | 1.60 | 0.59 |
| 3.category#3.seerstage | 0.03 | 0.02 | 1.60 | 0.78 |
| 3.category#4.seerstage | 0.05 | 0.05 | 1.00 | 0.84 |
| 1.surgery#2.seerstage | 0.15 | 0.17 | 6.00 | 0.30 |
| 1.surgery#3.seerstage | 0.07 | 0.07 | 2.70 | 0.66 |
| 1.surgery#4.seerstage | 0.07 | 0.07 | 3.30 | 0.54 |
| 2.surgery#3.seerstage | <0.01 | <0.01 | 6.70 | 0.08 |
| 2.surgery#4.seerstage | <0.01 | <0.01 | 3.10 | 0.56 |
| 1.radiation#2.seerstage | 0.08 | 0.09 | 2.40 | 0.48 |
| 1.radiation#3.seerstage | 0.12 | 0.11 | 3.40 | 0.57 |
| 1.radiation#4.seerstage | 0.04 | 0.04 | 2.20 | 0.64 |
| 2.radiation#2.seerstage | 0.16 | 0.17 | 1.70 | 0.67 |
| 2.radiation#3.seerstage | 0.25 | 0.25 | 0.90 | 0.88 |
| 2.radiation#4.seerstage | 0.08 | 0.08 | 2.50 | 0.62 |
| 1.radiation#1.surgery | 0.19 | 0.18 | 0.60 | 0.91 |
| 1.radiation#2.surgery | <0.01 | <0.01 | 3.40 | 0.32 |
| 2.radiation#1.surgery | 0.29 | 0.29 | 1.60 | 0.73 |
| 2.radiation#2.surgery | 0.01 | 0.01 | <0.01 | 1.00 |
| 2.category#1.surgery | 0.14 | 0.14 | 0.60 | 0.90 |
| 2.category#2.surgery | <0.01 | <0.01 | 5.00 | 0.16 |
| 3.category#1.surgery | 0.10 | 0.10 | 0.40 | 0.94 |
| 2.seerstage#c.age | 16.61 | 17.62 | 3.20 | 0.45 |
| 3.seerstage#c.age | 25.12 | 24.64 | 1.90 | 0.75 |
| 4.seerstage#c.age | 8.09 | 7.51 | 2.90 | 0.56 |
| 1.radiation#2.category | 0.09 | 0.09 | 1.20 | 0.76 |
| 1.radiation#3.category | 0.04 | 0.05 | 1.30 | 0.75 |
| 2.radiation#2.category | 0.19 | 0.19 | 1.00 | 0.82 |
| 2.radiation#3.category | 0.08 | 0.07 | 1.10 | 0.80 |
| 1.marital#2.seerstage | 0.11 | 0.11 | 1.80 | 0.62 |
| 1.marital#3.seerstage | 0.17 | 0.17 | 3.20 | 0.59 |
| 1.marital#4.seerstage | 0.06 | 0.06 | 2.80 | 0.58 |
| 2.marital#2.seerstage | 0.01 | 0.01 | 1.60 | 0.65 |
| 2.marital#3.seerstage | 0.02 | 0.01 | 7.30 | 0.16 |
| 2.marital#4.seerstage | 0.01 | <0.01 | 5.30 | 0.28 |
| 1.radiation#c.age | 20.89 | 20.41 | 1.50 | 0.73 |
| 2.radiation#c.age | 47.76 | 48.12 | 1.10 | 0.81 |
| 2.race#2.seerstage | 0.02 | 0.02 | 2.40 | 0.45 |
| 2.race#3.seerstage | 0.03 | 0.03 | 0.80 | 0.90 |
| 2.race#4.seerstage | 0.01 | 0.01 | 4.20 | 0.34 |
| 3.race#2.seerstage | 0.02 | 0.02 | 2.70 | 0.50 |
| 3.race#3.seerstage | 0.02 | 0.03 | 5.60 | 0.39 |
| 3.race#4.seerstage | 0.01 | 0.01 | 3.30 | 0.53 |
| 4.race#2.seerstage | 0.01 | 0.01 | <0.01 | 1.00 |
| 4.race#3.seerstage | 0.02 | 0.02 | 0.90 | 0.88 |
| 4.race#4.seerstage | <0.01 | <0.01 | 4.80 | 0.18 |
| 5.race#2.seerstage | <0.01 | <0.01 | <0.01 | 1.00 |
| 5.race#3.seerstage | <0.01 | <0.01 | 3.10 | 0.56 |
| 2.race#2.category | 0.02 | 0.02 | 1.10 | 0.77 |
| 2.race#3.category | 0.01 | 0.01 | 1.00 | 0.78 |
| 3.race#2.category | 0.01 | 0.01 | 2.90 | 0.47 |
| 4.race#2.category | 0.01 | 0.01 | <0.01 | 1.00 |
| 4.race#3.category | <0.01 | <0.01 | <0.01 | 1.00 |
| 5.race#2.category | <0.01 | <0.01 | <0.01 | 1.00 |
| 1.insurance#2.seerstage | 0.13 | 0.14 | 2.20 | 0.56 |
| 1.insurance#3.seerstage | 0.19 | 0.20 | 2.40 | 0.69 |
| 1.insurance#4.seerstage | 0.07 | 0.06 | 3.20 | 0.53 |
| 2.insurance#2.seerstage | 0.11 | 0.12 | 1.80 | 0.63 |
| 2.insurance#3.seerstage | 0.17 | 0.16 | 3.80 | 0.51 |
| 2.insurance#4.seerstage | 0.05 | 0.05 | 1.40 | 0.76 |
| 2.race#1.surgery | 0.04 | 0.03 | 0.60 | 0.90 |
| 2.race#2.surgery | <0.01 | <0.01 | 4.00 | 0.32 |
| 3.race#1.surgery | 0.02 | 0.02 | 0.80 | 0.88 |
| 3.race#2.surgery | <0.01 | <0.01 | <0.01 | 1.00 |
| 4.race#1.surgery | 0.01 | 0.01 | 2.90 | 0.56 |
| 4.race#2.surgery | <0.01 | <0.01 | <0.01 | 1.00 |
| 5.race#1.surgery | <0.01 | <0.01 | 3.70 | 0.56 |
| 2.race#1.insurance | 0.05 | 0.05 | 0.40 | 0.92 |
| 2.race#2.insurance | 0.03 | 0.03 | 0.90 | 0.80 |
| 3.race#1.insurance | 0.05 | 0.05 | 2.60 | 0.61 |
| 3.race#2.insurance | 0.01 | 0.01 | 5.00 | 0.22 |
| 4.race#1.insurance | 0.03 | 0.03 | 1.80 | 0.71 |
| 4.race#2.insurance | 0.02 | 0.02 | 0.80 | 0.86 |
| 5.race#1.insurance | <0.01 | <0.01 | 3.90 | 0.41 |
| 5.race#2.insurance | <0.01 | <0.01 | <0.01 | 1.00 |
| 2.sex#c.age | 67.68 | 67.49 | 0.70 | 0.76 |
| 2.race#c.age | 5.16 | 5.23 | 0.40 | 0.93 |
| 3.race#c.age | 4.21 | 4.18 | 0.20 | 0.97 |
| 4.race#c.age | 3.45 | 3.15 | 2.20 | 0.65 |
| 5.race#c.age | 0.31 | 0.17 | 3.70 | 0.41 |
| c.age#c.age | 4832.30 | 4807.20 | 1.70 | 0.70 |
| 1.marital#1.surgery | 0.24 | 0.23 | 1.80 | 0.72 |
| 1.marital#2.surgery | <0.01 | <0.01 | 1.80 | 0.74 |
| 2.marital#1.surgery | 0.02 | 0.01 | 7.50 | 0.10 |
| 2.race#1.marital | 0.02 | 0.02 | 1.70 | 0.61 |
| 2.race#2.marital | <0.01 | <0.01 | 1.40 | 0.71 |
| 3.race#1.marital | 0.03 | 0.03 | 1.80 | 0.70 |
| 3.race#2.marital | <0.01 | <0.01 | 1.90 | 0.71 |
| 4.race#1.marital | 0.02 | 0.02 | 0.70 | 0.88 |
| 4.race#2.marital | <0.01 | <0.01 | 2.50 | 0.56 |
| 5.race#1.marital | <0.01 | <0.01 | <0.01 | 1.00 |
| 1.radiation#2.race | 0.03 | 0.03 | 0.50 | 0.90 |
| 1.radiation#3.race | 0.02 | 0.02 | 0.80 | 0.88 |
| 1.radiation#4.race | 0.01 | 0.01 | 2.70 | 0.53 |
| 1.radiation#5.race | <0.01 | <0.01 | 3.30 | 0.32 |
| 2.radiation#2.race | 0.05 | 0.05 | 0.80 | 0.84 |
| 2.radiation#3.race | 0.04 | 0.04 | 1.10 | 0.82 |
| 2.radiation#4.race | 0.04 | 0.04 | 0.60 | 0.91 |
| 2.radiation#5.race | <0.01 | <0.01 | 2.00 | 0.71 |
| 2.seerstage#2.sex | 0.24 | 0.26 | 3.60 | 0.41 |
| 3.seerstage#2.sex | 0.36 | 0.35 | 1.70 | 0.78 |
| 4.seerstage#2.sex | 0.12 | 0.11 | 3.40 | 0.49 |
| 2.race#2.sex | 0.08 | 0.08 | 0.40 | 0.93 |
| 3.race#2.sex | 0.06 | 0.06 | 0.40 | 0.93 |
| 4.race#2.sex | 0.05 | 0.05 | 2.00 | 0.68 |
| 5.race#2.sex | <0.01 | <0.01 | 3.40 | 0.48 |
| 1.marital#1.insurance | 0.25 | 0.25 | 1.60 | 0.72 |
| 1.marital#2.insurance | 0.22 | 0.22 | 0.20 | 0.96 |
| 2.marital#1.insurance | 0.02 | 0.02 | 4.10 | 0.35 |
| 2.marital#2.insurance | 0.02 | 0.01 | 6.10 | 0.15 |
| 2.category#c.age | 19.12 | 18.88 | 0.70 | 0.87 |
| 3.category#c.age | 7.91 | 7.80 | 0.50 | 0.92 |
| 1.insurance#c.age | 37.93 | 38.78 | 2.40 | 0.59 |
| 2.insurance#c.age | 30.47 | 29.58 | 2.60 | 0.56 |
| 1.marital#2.category | 0.13 | 0.14 | 0.50 | 0.90 |
| 1.marital#3.category | 0.07 | 0.07 | 0.80 | 0.86 |
| 2.marital#2.category | 0.01 | 0.01 | 2.70 | 0.53 |
| 2.marital#3.category | <0.01 | <0.01 | 1.90 | 0.56 |

BCLA: breast duct carcinoma with subsequent primary lung adenocarcinoma; bias: absolutely standardized difference; p: t test between treated and control group

Tab S15 Balance test between group mTPC2 with BCLA and single lung cancer in matched cohort

| Variable | Mean | |  |  |
| --- | --- | --- | --- | --- |
|  | Treated | Control | bias | p |
| age | 73.12 | 73.33 | -1.80 | 0.84 |
| 2.seerstage | 0.17 | 0.17 | <0.01 | 1.00 |
| 3.seerstage | 0.06 | 0.06 | -3.60 | 0.81 |
| 4.seerstage | 0.14 | 0.13 | 2.10 | 0.87 |
| 1.radiation | 0.53 | 0.53 | 1.20 | 0.91 |
| 2.radiation | 0.47 | 0.48 | -1.20 | 0.91 |
| 1.surgery | 0.93 | 0.93 | <0.01 | 1.00 |
| 2.race | 0.11 | 0.11 | <0.01 | 1.00 |
| 3.race | 0.04 | 0.04 | -3.10 | 0.78 |
| 4.race | 0.04 | 0.04 | <0.01 | 1.00 |
| 5.race | 0.01 | 0.01 | <0.01 | 1.00 |
| 2.category | 0.25 | 0.25 | <0.01 | 1.00 |
| 3.category | 0.13 | 0.14 | -1.70 | 0.87 |
| 1.marital | 0.43 | 0.42 | 1.30 | 0.91 |
| 2.marital | 0.07 | 0.05 | 8.40 | 0.48 |
| 2.category#2.seerstage | 0.06 | 0.06 | <0.01 | 1.00 |
| 2.category#3.seerstage | 0.01 | 0.01 | <0.01 | 1.00 |
| 3.category#2.seerstage | 0.04 | 0.04 | <0.01 | 1.00 |
| 3.category#3.seerstage | 0.03 | 0.03 | -5.30 | 0.74 |
| c.age#c.age | 5430.60 | 5462.10 | -2.00 | 0.83 |
| 1.surgery#2.seerstage | 0.17 | 0.17 | <0.01 | 1.00 |
| 1.surgery#3.seerstage | 0.03 | 0.04 | -4.70 | 0.76 |
| 1.surgery#4.seerstage | 0.12 | 0.11 | 2.20 | 0.86 |
| 1.marital#2.seerstage | 0.07 | 0.06 | 3.20 | 0.82 |
| 1.marital#3.seerstage | 0.02 | 0.02 | <0.01 | 1.00 |
| 1.marital#4.seerstage | 0.05 | 0.05 | <0.01 | 1.00 |
| 2.marital#2.seerstage | 0.01 | 0.01 | <0.01 | 1.00 |
| 2.marital#4.seerstage | 0.03 | 0.01 | 16.20 | 0.18 |
| 1.marital#2.race | 0.01 | <0.01 | 4.30 | 0.32 |
| 1.marital#3.race | 0.02 | 0.02 | <0.01 | 1.00 |
| 1.marital#4.race | 0.03 | 0.03 | <0.01 | 1.00 |
| 1.marital#5.race | 0.01 | 0.01 | <0.01 | 1.00 |
| 2.marital#2.race | 0.01 | 0.01 | 6.70 | 0.56 |
| 2.marital#4.race | 0.01 | 0.01 | <0.01 | 1.00 |
| 2.race#2.seerstage | 0.04 | 0.03 | 4.40 | 0.76 |
| 2.race#3.seerstage | 0.01 | 0.01 | -10.20 | 0.56 |
| 2.race#4.seerstage | 0.03 | 0.03 | <0.01 | 1.00 |
| 3.race#2.seerstage | 0.01 | 0.01 | -10.30 | 0.56 |
| 3.race#4.seerstage | 0.01 | 0.01 | <0.01 | 1.00 |
| 4.race#4.seerstage | 0.01 | 0.01 | <0.01 | 1.00 |
| 2.category#2.race | 0.02 | 0.02 | <0.01 | 1.00 |
| 2.category#4.race | 0.01 | 0.01 | <0.01 | 1.00 |
| 3.category#2.race | 0.01 | 0.01 | <0.01 | 1.00 |
| 3.category#4.race | 0.01 | 0.01 | <0.01 | 1.00 |
| 2.race#1.radiation | 0.06 | 0.06 | <0.01 | 1.00 |
| 2.race#2.radiation | 0.04 | 0.04 | <0.01 | 1.00 |
| 3.race#1.radiation | 0.02 | 0.02 | <0.01 | 1.00 |
| 3.race#2.radiation | 0.02 | 0.03 | -4.40 | 0.70 |
| 4.race#1.radiation | 0.01 | 0.01 | <0.01 | 1.00 |
| 4.race#2.radiation | 0.04 | 0.04 | <0.01 | 1.00 |
| 5.race#1.radiation | 0.01 | 0.01 | <0.01 | 1.00 |
| 2.race#c.age | 7.76 | 7.82 | -0.30 | 0.98 |
| 3.race#c.age | 2.65 | 3.20 | -4.30 | 0.73 |
| 4.race#c.age | 3.04 | 3.04 | <0.01 | 1.00 |
| 5.race#c.age | 0.51 | 0.51 | <0.01 | 1.00 |
| 2.category#c.age | 18.11 | 18.11 | <0.01 | 1.00 |
| 3.category#c.age | 9.34 | 9.77 | -1.70 | 0.88 |
| 1.surgery#1.radiation | 0.51 | 0.50 | 1.20 | 0.91 |
| 1.surgery#2.radiation | 0.43 | 0.43 | -1.30 | 0.91 |
| 1.marital#2.category | 0.09 | 0.09 | <0.01 | 1.00 |
| 1.marital#3.category | 0.06 | 0.07 | -2.30 | 0.82 |
| 2.marital#2.category | 0.03 | 0.03 | <0.01 | 1.00 |
| 2.marital#3.category | 0.01 | 0.01 | <0.01 | 1.00 |
| 1.surgery#c.age | 68.14 | 68.31 | -0.90 | 0.94 |
| 2.race#1.surgery | 0.09 | 0.09 | 2.10 | 0.85 |
| 3.race#1.surgery | 0.04 | 0.04 | -3.10 | 0.78 |
| 4.race#1.surgery | 0.04 | 0.04 | <0.01 | 1.00 |
| 5.race#1.surgery | 0.01 | 0.01 | <0.01 | 1.00 |

BCLA: breast duct carcinoma with subsequent primary lung adenocarcinoma; bias: absolutely standardized difference; p: t test between treated and control group

Tab S16 Balance test between patients with LABC and single lung cancer in matched cohort

| Variable | Mean | |  |  |
| --- | --- | --- | --- | --- |
|  | Treated | Control | bias | p |
| age | 66.32 | 66.40 | 0.7 | 0.90 |
| 2.sex | 0.99 | 0.99 | 0 | 1.00 |
| 1.surgery | 0.68 | 0.68 | 0.4 | 0.95 |
| 2.surgery | <0.01 | <0.01 | 2.6 | 0.56 |
| 2.seerstage | 0.24 | 0.24 | 0.4 | 0.95 |
| 3.seerstage | 0.22 | 0.23 | 0.6 | 0.94 |
| 4.seerstage | 0.14 | 0.14 | 0.5 | 0.93 |
| 2.race | 0.12 | 0.13 | 1.1 | 0.86 |
| 3.race | 0.05 | 0.05 | 0.7 | 0.89 |
| 4.race | 0.03 | 0.03 | 2.5 | 0.62 |
| 5.race | 0.01 | <0.01 | 7.7 | 0.08 |
| 1.marital | 0.47 | 0.47 | 1.1 | 0.86 |
| 2.marital | 0.04 | 0.04 | 2.7 | 0.66 |
| 2.seerstage#1.surgery | 0.20 | 0.20 | 0.6 | 0.94 |
| 3.seerstage#1.surgery | 0.06 | 0.06 | 0 | 1.00 |
| 4.seerstage#1.surgery | 0.11 | 0.12 | 1.3 | 0.85 |
| 4.seerstage#2.surgery | <0.01 | <0.01 | 0 | 1.00 |
| 2.seerstage#c.age | 16.19 | 16.08 | 0.3 | 0.95 |
| 3.seerstage#c.age | 14.57 | 14.77 | 1 | 0.90 |
| 4.seerstage#c.age | 9.26 | 9.34 | 0.4 | 0.95 |
| c.age#c.age | 4505.20 | 4511.90 | 0.5 | 0.93 |
| 1.surgery#c.age | 44.13 | 44.24 | 0.4 | 0.95 |
| 2.surgery#c.age | 0.25 | 0.13 | 2.6 | 0.58 |
| 2.race#2.seerstage | 0.03 | 0.03 | 3.4 | 0.50 |
| 2.race#3.seerstage | 0.03 | 0.03 | 2.6 | 0.74 |
| 2.race#4.seerstage | 0.02 | 0.02 | 0 | 1.00 |
| 3.race#2.seerstage | 0.01 | 0.01 | 0 | 1.00 |
| 3.race#3.seerstage | 0.01 | 0.01 | 2.1 | 0.80 |
| 3.race#4.seerstage | <0.01 | <0.01 | 0 | 1.00 |
| 4.race#2.seerstage | 0.01 | 0.01 | 1.2 | 0.74 |
| 4.race#3.seerstage | 0.02 | 0.01 | 5.7 | 0.44 |
| 4.race#4.seerstage | <0.01 | <0.01 | 2.8 | 0.56 |
| 5.race#2.seerstage | <0.01 | <0.01 | 3.9 | 0.32 |
| 5.race#4.seerstage | <0.01 | <0.01 | 5.1 | 0.32 |
| 1.marital#2.race | 0.04 | 0.05 | 2.7 | 0.66 |
| 1.marital#3.race | 0.03 | 0.03 | 0.9 | 0.86 |
| 1.marital#4.race | 0.02 | 0.02 | 0 | 1.00 |
| 1.marital#5.race | <0.01 | <0.01 | 4 | 0.32 |
| 2.marital#2.race | <0.01 | <0.01 | 2.8 | 0.32 |
| 2.marital#3.race | <0.01 | <0.01 | 0 | 1.00 |
| 2.sex#c.age | 65.54 | 65.61 | 0.3 | 0.92 |
| 1.marital#1.surgery | 0.35 | 0.35 | 1.2 | 0.85 |
| 2.marital#1.surgery | 0.02 | 0.02 | 2.8 | 0.69 |
| 2.marital#2.surgery | <0.01 | <0.01 | 5 | 0.32 |
| 1.marital#2.seerstage | 0.12 | 0.12 | 1.3 | 0.79 |
| 1.marital#3.seerstage | 0.09 | 0.09 | 0.8 | 0.92 |
| 1.marital#4.seerstage | 0.07 | 0.07 | 0.7 | 0.91 |
| 2.marital#2.seerstage | 0.01 | 0.01 | 3 | 0.53 |
| 2.marital#3.seerstage | 0.01 | 0.01 | 0 | 1.00 |
| 2.marital#4.seerstage | <0.01 | <0.01 | 0 | 1.00 |

BCLA: breast duct carcinoma with subsequent primary lung adenocarcinoma; bias: absolutely standardized difference; p: t test between treated and control group

Tab S17 Balance test between group sTPC with LABC and single lung cancer in matched cohort

| Variable | Mean | |  |  |
| --- | --- | --- | --- | --- |
|  | Treated | Control | bias | p |
| age | 68.39 | 68.48 | 0.80 | 0.93 |
| 2.seerstage | 0.19 | 0.19 | 1.10 | 0.90 |
| 3.seerstage | 0.45 | 0.46 | 1.30 | 0.92 |
| 4.seerstage | 0.14 | 0.13 | 1.40 | 0.89 |
| 2.sex | 1.00 | 1.00 | <0.01 | 1.00 |
| 1.surgery | 0.40 | 0.39 | 2.10 | 0.84 |
| 1.radiation | 0.52 | 0.53 | 1.00 | 0.92 |
| 2.radiation | 0.48 | 0.47 | 1.00 | 0.92 |
| 2.category | 0.25 | 0.27 | 4.30 | 0.66 |
| 3.category | 0.14 | 0.13 | 2.60 | 0.77 |
| 2.race | 0.11 | 0.08 | 9.20 | 0.31 |
| 3.race | 0.06 | 0.06 | 1.90 | 0.84 |
| 4.race | 0.04 | 0.04 | <0.01 | 1.00 |
| 5.race | <0.01 | <0.01 | 9.70 | 0.32 |
| 1.insurance | 0.55 | 0.54 | 1.90 | 0.84 |
| 2.insurance | 0.44 | 0.46 | 2.90 | 0.77 |
| 1.surgery#2.seerstage | 0.13 | 0.14 | 1.80 | 0.89 |
| 1.surgery#3.seerstage | 0.08 | 0.07 | 4.80 | 0.71 |
| 1.surgery#4.seerstage | 0.08 | 0.08 | <0.01 | 1.00 |
| 2.category#1.radiation | 0.11 | 0.13 | 4.50 | 0.65 |
| 2.category#2.radiation | 0.14 | 0.14 | 1.30 | 0.89 |
| 3.category#1.radiation | 0.03 | 0.03 | 2.00 | 0.78 |
| 3.category#2.radiation | 0.10 | 0.10 | 1.60 | 0.87 |
| 2.seerstage#c.age | 12.93 | 13.10 | 0.50 | 0.95 |
| 3.seerstage#c.age | 3<0.01 | 30.65 | 2.60 | 0.84 |
| 4.seerstage#c.age | 9.39 | 9.00 | 1.60 | 0.87 |
| 2.category#2.seerstage | 0.06 | 0.06 | <0.01 | 1.00 |
| 2.category#3.seerstage | 0.15 | 0.17 | 7.30 | 0.59 |
| 3.category#2.seerstage | 0.02 | 0.02 | <0.01 | 1.00 |
| 3.category#3.seerstage | 0.01 | <0.01 | 9.50 | 0.32 |
| 3.category#4.seerstage | 0.07 | 0.07 | <0.01 | 1.00 |
| 2.race#2.seerstage | 0.01 | 0.01 | <0.01 | 1.00 |
| 2.race#3.seerstage | 0.07 | 0.04 | 15.50 | 0.21 |
| 2.race#4.seerstage | 0.01 | 0.01 | <0.01 | 1.00 |
| 3.race#2.seerstage | <0.01 | <0.01 | <0.01 | 1.00 |
| 3.race#3.seerstage | 0.03 | 0.03 | 3.90 | 0.78 |
| 3.race#4.seerstage | <0.01 | <0.01 | <0.01 | 1.00 |
| 4.race#2.seerstage | <0.01 | <0.01 | <0.01 | 1.00 |
| 4.race#3.seerstage | 0.02 | 0.03 | 4.30 | 0.76 |
| 5.race#4.seerstage | <0.01 | <0.01 | 9.70 | 0.32 |
| 1.insurance#2.race | 0.04 | 0.01 | 15.60 | 0.03 |
| 1.insurance#3.race | 0.05 | 0.06 | 4.60 | 0.66 |
| 1.insurance#4.race | 0.02 | 0.02 | <0.01 | 1.00 |
| 1.insurance#5.race | <0.01 | <0.01 | 9.70 | 0.32 |
| 2.insurance#2.race | 0.05 | 0.06 | 4.20 | 0.68 |
| 2.insurance#3.race | 0.01 | <0.01 | 3.20 | 0.56 |
| 2.insurance#4.race | 0.02 | 0.02 | <0.01 | 1.00 |
| 1.radiation#2.seerstage | 0.12 | 0.12 | 1.30 | 0.88 |
| 1.radiation#3.seerstage | 0.25 | 0.26 | 3.10 | 0.82 |
| 1.radiation#4.seerstage | 0.06 | 0.06 | 2.00 | 0.84 |
| 2.radiation#2.seerstage | 0.07 | 0.07 | <0.01 | 1.00 |
| 2.radiation#3.seerstage | 0.21 | 0.20 | 1.60 | 0.90 |
| 2.radiation#4.seerstage | 0.07 | 0.07 | <0.01 | 1.00 |
| 2.race#2.category | 0.05 | 0.06 | 4.90 | 0.66 |
| 2.race#3.category | <0.01 | <0.01 | <0.01 | 1.00 |
| 3.race#2.category | <0.01 | <0.01 | <0.01 | 1.00 |
| 3.race#3.category | <0.01 | <0.01 | 5.40 | 0.32 |
| 4.race#2.category | 0.01 | 0.01 | <0.01 | 1.00 |
| 2.race#1.radiation | 0.06 | 0.03 | 12.50 | 0.17 |
| 2.race#2.radiation | 0.04 | 0.04 | <0.01 | 1.00 |
| 3.race#1.radiation | <0.01 | <0.01 | <0.01 | 1.00 |
| 3.race#2.radiation | 0.05 | 0.06 | 2.20 | 0.83 |
| 4.race#1.radiation | 0.03 | 0.03 | 3.30 | 0.78 |
| 4.race#2.radiation | 0.01 | 0.01 | 3.30 | 0.65 |
| 5.race#1.radiation | <0.01 | <0.01 | 9.70 | 0.32 |
| 1.radiation#1.surgery | 0.20 | 0.20 | 1.50 | 0.90 |
| 2.radiation#1.surgery | 0.19 | 0.19 | 1.20 | 0.90 |
| 2.category#1.surgery | 0.09 | 0.09 | 1.70 | 0.86 |
| 3.category#1.surgery | 0.12 | 0.11 | 3.40 | 0.76 |
| c.age#c.age | 4790.90 | 4796.30 | 0.40 | 0.97 |
| 2.race#c.age | 6.41 | 4.55 | 9.40 | 0.28 |
| 3.race#c.age | 4.21 | 4.53 | 1.80 | 0.85 |
| 4.race#c.age | 2.94 | 2.85 | 0.70 | 0.95 |
| 5.race#c.age | 0.38 | <0.01 | 9.80 | 0.32 |

BCLA: breast duct carcinoma with subsequent primary lung adenocarcinoma; bias: absolutely standardized difference; p: t test between treated and control group

Tab S18 Balance test between group mTPC1 with LABC and single lung cancer in matched cohort

|  | Mean | |  |  |
| --- | --- | --- | --- | --- |
| Variable | Treated | Control | bias | p |
| age | 66.88 | 66.71 | 1.70 | 0.83 |
| 2.sex | 0.98 | 0.98 | <0.01 | 1.00 |
| 1.radiation | 0.55 | 0.55 | 0.70 | 0.93 |
| 2.radiation | 0.43 | 0.43 | 0.70 | 0.93 |
| 2.race | 0.13 | 0.14 | 1.20 | 0.90 |
| 3.race | 0.04 | 0.04 | <0.01 | 1.00 |
| 4.race | 0.03 | 0.03 | <0.01 | 1.00 |
| 5.race | 0.01 | <0.01 | 5.70 | 0.56 |
| 1.insurance | 0.42 | 0.40 | 3.60 | 0.67 |
| 2.insurance | 0.57 | 0.59 | 4.30 | 0.61 |
| c.age#c.age | 4568.90 | 4549.30 | 1.40 | 0.86 |
| 1.insurance#2.race | 0.06 | 0.05 | 3.50 | 0.71 |
| 1.insurance#3.race | 0.02 | 0.02 | <0.01 | 1.00 |
| 1.insurance#4.race | 0.02 | 0.02 | <0.01 | 1.00 |
| 2.insurance#2.race | 0.07 | 0.08 | 5.00 | 0.64 |
| 2.insurance#3.race | 0.02 | 0.02 | <0.01 | 1.00 |
| 2.insurance#4.race | 0.01 | 0.01 | <0.01 | 1.00 |
| 2.insurance#5.race | 0.01 | <0.01 | 5.70 | 0.56 |
| 2.sex#c.age | 65.56 | 65.39 | 0.70 | 0.88 |
| 2.race#2.sex | 0.13 | 0.14 | 1.20 | 0.90 |
| 3.race#2.sex | 0.04 | 0.04 | <0.01 | 1.00 |
| 4.race#2.sex | 0.03 | 0.03 | <0.01 | 1.00 |
| 5.race#2.sex | 0.01 | <0.01 | 5.70 | 0.56 |

LABC: breast duct carcinoma with prior primary lung adenocarcinoma bias: absolutely standardized difference; p: t test between treated and control group

Tab S19 Balance test between group mTPC2 with LABC and single lung cancer in matched cohort

| Variable | Mean | |  |  |
| --- | --- | --- | --- | --- |
|  | Treated | Control | bias | p |
| age | 63.50 | 63.49 | 0.10 | 1.00 |
| 2.sex | 0.99 | 0.99 | <0.01 | 1.00 |
| 2.seerstage | 0.25 | 0.25 | 1.70 | 0.90 |
| 3.seerstage | 0.03 | 0.03 | <0.01 | 1.00 |
| 4.seerstage | 0.19 | 0.19 | <0.01 | 1.00 |
| 1.surgery | 0.94 | 0.94 | <0.01 | 1.00 |
| 2.category | 0.45 | 0.45 | 1.30 | 0.91 |
| 3.category | 0.39 | 0.41 | 2.80 | 0.82 |
| 1.radiation | 0.37 | 0.39 | 5.60 | 0.64 |
| 2.radiation | 0.63 | 0.61 | 5.60 | 0.64 |
| 2.race | 0.11 | 0.09 | 6.40 | 0.57 |
| 3.race | 0.04 | 0.03 | 2.90 | 0.76 |
| 4.race | 0.03 | 0.01 | 6.20 | 0.41 |
| 1.insurance | 0.13 | 0.12 | 1.50 | 0.87 |
| 2.insurance | 0.87 | 0.88 | 1.50 | 0.87 |
| 2.category#2.seerstage | 0.13 | 0.14 | 4.60 | 0.74 |
| 2.category#3.seerstage | 0.01 | 0.01 | 6.10 | 0.56 |
| 3.category#2.seerstage | 0.07 | 0.07 | <0.01 | 1.00 |
| 3.category#3.seerstage | 0.01 | 0.02 | 18.10 | 0.32 |
| 3.category#4.seerstage | 0.19 | 0.19 | <0.01 | 1.00 |
| 1.radiation#2.category | 0.21 | 0.24 | 7.40 | 0.59 |
| 1.radiation#3.category | 0.05 | 0.05 | <0.01 | 1.00 |
| 2.radiation#2.category | 0.24 | 0.22 | 4.30 | 0.69 |
| 2.radiation#3.category | 0.34 | 0.36 | 3.00 | 0.81 |
| 2.race#2.seerstage | 0.03 | 0.02 | 8.90 | 0.48 |
| 2.race#4.seerstage | 0.01 | 0.01 | <0.01 | 1.00 |
| 3.race#2.seerstage | 0.01 | 0.01 | 5.60 | 0.56 |
| 4.race#2.seerstage | 0.01 | 0.01 | <0.01 | 1.00 |
| 4.race#4.seerstage | 0.01 | 0.01 | <0.01 | 1.00 |
| 1.radiation#2.seerstage | 0.12 | 0.14 | 6.90 | 0.62 |
| 1.radiation#3.seerstage | 0.02 | 0.02 | <0.01 | 1.00 |
| 1.radiation#4.seerstage | 0.01 | 0.01 | <0.01 | 1.00 |
| 2.radiation#2.seerstage | 0.13 | 0.11 | 8.70 | 0.49 |
| 2.radiation#3.seerstage | 0.01 | 0.01 | <0.01 | 1.00 |
| 2.radiation#4.seerstage | 0.18 | 0.18 | <0.01 | 1.00 |
| 1.radiation#c.age | 24.00 | 25.58 | 5.40 | 0.66 |
| 2.radiation#c.age | 39.50 | 37.91 | 5.30 | 0.66 |
| 2.seerstage#c.age | 16.17 | 15.85 | 1.30 | 0.92 |
| 3.seerstage#c.age | 1.34 | 1.17 | 1.70 | 0.85 |
| 4.seerstage#c.age | 11.61 | 11.61 | <0.01 | 1.00 |
| 2.race#2.category | 0.07 | 0.06 | 2.90 | 0.82 |
| 2.race#3.category | 0.02 | 0.02 | <0.01 | 1.00 |
| 3.race#2.category | 0.03 | 0.03 | 3.90 | 0.74 |
| 4.race#2.category | 0.01 | <0.01 | 5.80 | 0.32 |
| 4.race#3.category | 0.01 | 0.01 | 5.80 | 0.56 |
| 2.race#1.radiation | 0.03 | 0.03 | 3.90 | 0.74 |
| 2.race#2.radiation | 0.08 | 0.06 | 5.00 | 0.66 |
| 3.race#1.radiation | 0.01 | 0.01 | <0.01 | 1.00 |
| 3.race#2.radiation | 0.03 | 0.03 | 3.20 | 0.74 |
| 4.race#1.radiation | 0.01 | 0.01 | 4.50 | 0.56 |
| 4.race#2.radiation | 0.01 | 0.01 | 4.10 | 0.56 |
| c.age#c.age | 4123.90 | 4127.20 | 0.30 | 0.98 |
| 1.insurance#2.race | 0.02 | 0.01 | 7.90 | 0.32 |
| 1.insurance#3.race | 0.01 | <0.01 | 8.80 | 0.16 |
| 1.insurance#4.race | 0.01 | 0.01 | <0.01 | 1.00 |
| 2.insurance#2.race | 0.09 | 0.08 | 2.50 | 0.84 |
| 2.insurance#3.race | 0.03 | 0.03 | 3.70 | 0.74 |
| 2.insurance#4.race | 0.02 | 0.01 | 7.90 | 0.32 |
| 1.insurance#2.seerstage | 0.04 | 0.03 | 9.20 | 0.36 |
| 1.insurance#3.seerstage | 0.01 | <0.01 | 5.50 | 0.32 |
| 2.insurance#2.seerstage | 0.21 | 0.22 | 3.80 | 0.79 |
| 2.insurance#3.seerstage | 0.02 | 0.03 | 5.00 | 0.70 |
| 2.insurance#4.seerstage | 0.19 | 0.19 | <0.01 | 1.00 |

LABC: breast duct carcinoma with prior primary lung adenocarcinoma bias: absolutely standardized difference; p: t test between treated and control group

Tab S20 Balance test between patients with LABC and single breast cancer in matched cohort

| Variable | Mean | |  |  |
| --- | --- | --- | --- | --- |
|  | Treated | Control | bias | p |
| age | 70.18 | 70.41 | 1.8 | 0.71 |
| 2.seerstage | 0.16 | 0.16 | 0 | 1.00 |
| 3.seerstage | 0.07 | 0.07 | 0.9 | 0.91 |
| 4.seerstage | 0.10 | 0.10 | 0.7 | 0.92 |
| 1.radiation | 0.44 | 0.44 | 0.4 | 0.95 |
| 2.radiation | 0.56 | 0.56 | 0.4 | 0.95 |
| 1.surgery | 0.78 | 0.78 | 0 | 1.00 |
| 2.surgery | 0.01 | 0.01 | 0 | 1.00 |
| 2.race | 0.12 | 0.12 | 1.1 | 0.86 |
| 3.race | 0.05 | 0.05 | 0 | 1.00 |
| 4.race | 0.04 | 0.03 | 0.7 | 0.87 |
| 5.race | 0.01 | 0.01 | 4.3 | 0.48 |
| 2.category | 0.24 | 0.24 | 0.4 | 0.95 |
| 3.category | 0.13 | 0.13 | 0 | 1.00 |
| 1.surgery#2.seerstage | 0.15 | 0.15 | 0 | 1.00 |
| 1.surgery#3.seerstage | 0.04 | 0.04 | 0 | 1.00 |
| 1.surgery#4.seerstage | 0.06 | 0.06 | 0 | 1.00 |
| c.age#c.age | 5029.60 | 5063.40 | 2.1 | 0.69 |
| 2.category#2.seerstage | 0.04 | 0.04 | 0 | 1.00 |
| 2.category#3.seerstage | 0.02 | 0.02 | 1.6 | 0.84 |
| 3.category#2.seerstage | 0.03 | 0.03 | 0 | 1.00 |
| 3.category#3.seerstage | 0.02 | 0.02 | 0 | 1.00 |
| 1.surgery#c.age | 54.99 | 55.04 | 0.2 | 0.98 |
| 2.surgery#c.age | 0.38 | 0.38 | 0 | 1.00 |
| 2.race#2.seerstage | 0.02 | 0.02 | 0 | 1.00 |
| 2.race#3.seerstage | 0.02 | 0.02 | 0 | 1.00 |
| 2.race#4.seerstage | 0.02 | 0.01 | 3.3 | 0.64 |
| 3.race#2.seerstage | 0.01 | 0.01 | 0 | 1.00 |
| 3.race#4.seerstage | <0.01 | <0.01 | 0 | 1.00 |
| 4.race#2.seerstage | <0.01 | <0.01 | 0 | 1.00 |
| 4.race#3.seerstage | 0.01 | <0.01 | 3.1 | 0.65 |
| 4.race#4.seerstage | <0.01 | <0.01 | 0 | 1.00 |
| 5.race#2.seerstage | <0.01 | <0.01 | 0 | 1.00 |
| 5.race#4.seerstage | <0.01 | 0.01 | 9.7 | 0.32 |
| 2.category#c.age | 16.95 | 17.12 | 0.6 | 0.93 |
| 3.category#c.age | 9.01 | 9.01 | 0 | 1.00 |
| 2.category#1.surgery | 0.20 | 0.20 | 0 | 1.00 |
| 2.category#2.surgery | <0.01 | <0.01 | 0 | 1.00 |
| 3.category#1.surgery | 0.13 | 0.13 | 0 | 1.00 |
| 1.radiation#2.seerstage | 0.07 | 0.07 | 0 | 1.00 |
| 1.radiation#3.seerstage | 0.03 | 0.03 | 1.5 | 0.86 |
| 1.radiation#4.seerstage | 0.04 | 0.04 | 0 | 1.00 |
| 2.radiation#2.seerstage | 0.09 | 0.09 | 0 | 1.00 |
| 2.radiation#3.seerstage | 0.05 | 0.05 | 0 | 1.00 |
| 2.radiation#4.seerstage | 0.06 | 0.06 | 0.9 | 0.90 |
| 2.race#c.age | 8.12 | 7.92 | 1 | 0.87 |
| 3.race#c.age | 3.31 | 3.31 | 0 | 1.00 |
| 4.race#c.age | 2.47 | 2.35 | 0.8 | 0.87 |
| 5.race#c.age | 0.40 | 0.78 | 7.1 | 0.36 |
| 1.surgery#1.radiation | 0.39 | 0.39 | 0 | 1.00 |
| 1.surgery#2.radiation | 0.39 | 0.39 | 0 | 1.00 |
| 2.surgery#1.radiation | <0.01 | <0.01 | 0 | 1.00 |
| 2.surgery#2.radiation | <0.01 | <0.01 | 0 | 1.00 |
| 2.category#1.radiation | 0.10 | 0.10 | 0.5 | 0.92 |
| 2.category#2.radiation | 0.14 | 0.14 | 0 | 1.00 |
| 3.category#1.radiation | 0.06 | 0.06 | 0 | 1.00 |
| 3.category#2.radiation | 0.07 | 0.07 | 0 | 1.00 |
| 2.category#2.race | 0.03 | 0.03 | 0 | 1.00 |
| 2.category#3.race | 0.01 | 0.01 | 0 | 1.00 |
| 2.category#4.race | 0.01 | 0.01 | 0 | 1.00 |
| 2.category#5.race | <0.01 | <0.01 | 0 | 1.00 |
| 3.category#2.race | 0.01 | 0.01 | 0 | 1.00 |
| 3.category#3.race | <0.01 | <0.01 | 0 | 1.00 |
| 3.category#4.race | <0.01 | <0.01 | 0 | 1.00 |
| 2.seerstage#c.age | 11.25 | 11.29 | 0.2 | 0.98 |
| 3.seerstage#c.age | 5.00 | 4.93 | 0.5 | 0.95 |
| 4.seerstage#c.age | 6.90 | 7.16 | 1.4 | 0.84 |
| 1.radiation#c.age | 30.39 | 30.45 | 0.2 | 0.97 |
| 2.radiation#c.age | 39.79 | 39.95 | 0.5 | 0.94 |

LABC: breast duct carcinoma with prior primary lung adenocarcinoma; bias: absolutely standardized difference; p: t test between treated and control group

Tab S21 Balance test between group sTPC with LABC and single breast cancer in matched cohort

| Variable | Mean | |  |  |
| --- | --- | --- | --- | --- |
|  | Treated | Control | bias | p |
| age | 68.41 | 68.29 | 1.00 | 0.91 |
| 1.surgery | 0.58 | 0.59 | 1.20 | 0.92 |
| 2.surgery | 0.01 | <0.01 | 6.40 | 0.56 |
| 1.radiation | 0.37 | 0.37 | 1.00 | 0.92 |
| 2.radiation | 0.63 | 0.63 | 1.00 | 0.92 |
| 2.seerstage | 0.12 | 0.11 | 1.70 | 0.88 |
| 3.seerstage | 0.10 | 0.11 | 6.50 | 0.64 |
| 4.seerstage | 0.08 | 0.08 | <0.01 | 1.00 |
| 2.race | 0.11 | 0.11 | 3.10 | 0.76 |
| 3.race | 0.06 | 0.06 | 1.90 | 0.84 |
| 4.race | 0.04 | 0.04 | 2.00 | 0.81 |
| 1.insurance | 0.55 | 0.54 | 1.00 | 0.92 |
| 2.insurance | 0.44 | 0.46 | 2.90 | 0.77 |
| 1.marital | 0.41 | 0.39 | 2.90 | 0.77 |
| 2.marital | 0.07 | 0.06 | 4.40 | 0.69 |
| 2.seerstage#1.surgery | 0.10 | 0.09 | 2.00 | 0.87 |
| 3.seerstage#1.surgery | 0.03 | 0.04 | 7.50 | 0.59 |
| 4.seerstage#1.surgery | 0.02 | 0.02 | <0.01 | 1.00 |
| c.age#c.age | 4795.00 | 4777.60 | 1.10 | 0.90 |
| 1.insurance#2.seerstage | 0.06 | 0.06 | <0.01 | 1.00 |
| 1.insurance#3.seerstage | 0.05 | 0.05 | <0.01 | 1.00 |
| 1.insurance#4.seerstage | 0.06 | 0.06 | <0.01 | 1.00 |
| 2.insurance#2.seerstage | 0.05 | 0.05 | <0.01 | 1.00 |
| 2.insurance#3.seerstage | 0.05 | 0.06 | 8.90 | 0.52 |
| 2.insurance#4.seerstage | 0.02 | 0.02 | 4.80 | 0.74 |
| 2.race#c.age | 6.37 | 6.83 | 2.50 | 0.81 |
| 3.race#c.age | 4.18 | 4.53 | 2.20 | 0.84 |
| 4.race#c.age | 2.91 | 2.58 | 2.20 | 0.80 |
| 2.race#2.seerstage | 0.01 | 0.01 | 4.50 | 0.65 |
| 2.race#3.seerstage | 0.02 | 0.04 | 23.90 | 0.16 |
| 2.race#4.seerstage | 0.01 | 0.01 | <0.01 | 1.00 |
| 3.race#2.seerstage | 0.01 | 0.01 | <0.01 | 1.00 |
| 3.race#4.seerstage | <0.01 | <0.01 | <0.01 | 1.00 |
| 4.race#2.seerstage | <0.01 | <0.01 | <0.01 | 1.00 |
| 4.race#3.seerstage | 0.01 | <0.01 | 6.70 | 0.56 |
| 1.marital#2.seerstage | 0.06 | 0.06 | <0.01 | 1.00 |
| 1.marital#3.seerstage | 0.03 | 0.02 | 7.10 | 0.56 |
| 1.marital#4.seerstage | 0.01 | 0.01 | <0.01 | 1.00 |
| 2.marital#2.seerstage | <0.01 | <0.01 | <0.01 | 1.00 |
| 2.marital#4.seerstage | 0.01 | 0.01 | <0.01 | 1.00 |
| 1.insurance#2.race | 0.04 | 0.05 | 4.60 | 0.63 |
| 1.insurance#3.race | 0.05 | 0.05 | 2.30 | 0.82 |
| 1.insurance#4.race | 0.02 | 0.02 | 2.60 | 0.74 |
| 2.insurance#2.race | 0.06 | 0.07 | 4.20 | 0.69 |
| 2.insurance#3.race | 0.01 | 0.01 | <0.01 | 1.00 |
| 2.insurance#4.race | 0.02 | 0.02 | <0.01 | 1.00 |
| 2.seerstage#c.age | 8.17 | 7.93 | 1.30 | 0.91 |
| 3.seerstage#c.age | 6.35 | 7.15 | 5.60 | 0.68 |
| 4.seerstage#c.age | 5.74 | 5.92 | 1.00 | 0.93 |
| 1.marital#2.race | 0.03 | 0.03 | 2.60 | 0.78 |
| 1.marital#3.race | 0.04 | 0.04 | <0.01 | 1.00 |
| 1.marital#4.race | 0.02 | 0.01 | 2.70 | 0.70 |
| 2.marital#2.race | 0.01 | <0.01 | 5.70 | 0.56 |
| 2.race#1.surgery | 0.03 | 0.04 | 2.00 | 0.79 |
| 2.race#2.surgery | <0.01 | <0.01 | 9.50 | 0.32 |
| 3.race#1.surgery | 0.03 | 0.03 | <0.01 | 1.00 |
| 4.race#1.surgery | 0.02 | 0.02 | <0.01 | 1.00 |
| 2.seerstage#1.radiation | 0.06 | 0.06 | <0.01 | 1.00 |
| 2.seerstage#2.radiation | 0.06 | 0.05 | 2.30 | 0.83 |
| 3.seerstage#1.radiation | 0.04 | 0.05 | 10.40 | 0.48 |
| 3.seerstage#2.radiation | 0.06 | 0.06 | <0.01 | 1.00 |
| 4.seerstage#1.radiation | 0.02 | 0.02 | 3.10 | 0.74 |
| 4.seerstage#2.radiation | 0.06 | 0.06 | 2.40 | 0.84 |
| 1.surgery#c.age | 40.15 | 40.42 | 1.00 | 0.94 |
| 2.surgery#c.age | 0.67 | 0.31 | 7.00 | 0.53 |
| 1.insurance#c.age | 38.25 | 37.89 | 1.10 | 0.92 |
| 2.insurance#c.age | 29.64 | 30.41 | 2.30 | 0.82 |
| 1.insurance#1.surgery | 0.26 | 0.27 | 1.00 | 0.91 |
| 1.insurance#2.surgery | <0.01 | <0.01 | <0.01 | 1.00 |
| 2.insurance#1.surgery | 0.32 | 0.32 | 1.00 | 0.92 |
| 2.insurance#2.surgery | <0.01 | <0.01 | 9.20 | 0.32 |
| 1.marital#1.surgery | 0.28 | 0.28 | <0.01 | 1.00 |
| 1.marital#2.surgery | <0.01 | <0.01 | <0.01 | 1.00 |
| 2.marital#1.surgery | 0.02 | 0.02 | <0.01 | 1.00 |
| 2.marital#2.surgery | <0.01 | <0.01 | 9.50 | 0.32 |

LABC: breast duct carcinoma with prior primary lung adenocarcinoma; bias: absolutely standardized difference; p: t test between treated and control group

Tab S22 Balance test between group mTPC1 with LABC and single breast cancer in matched cohort

|  | Mean | |  |  |
| --- | --- | --- | --- | --- |
| Variable | Treated | Control | bias | p |
| age | 69.35 | 69.37 | 0.10 | 0.99 |
| 2.seerstage | 0.21 | 0.21 | 1.10 | 0.92 |
| 3.seerstage | 0.07 | 0.07 | 1.90 | 0.87 |
| 4.seerstage | 0.08 | 0.08 | 1.50 | 0.88 |
| 1.radiation | 0.44 | 0.44 | 0.70 | 0.93 |
| 2.radiation | 0.56 | 0.56 | 0.70 | 0.93 |
| 2.race | 0.14 | 0.13 | 1.10 | 0.90 |
| 3.race | 0.04 | 0.04 | 1.60 | 0.82 |
| 4.race | 0.03 | 0.03 | <0.01 | 1.00 |
| 5.race | 0.01 | 0.01 | <0.01 | 1.00 |
| 1.surgery | 0.87 | 0.87 | 1.30 | 0.90 |
| 2.surgery | <0.01 | <0.01 | <0.01 | 1.00 |
| 2.category | 0.27 | 0.27 | <0.01 | 1.00 |
| 3.category | 0.13 | 0.13 | <0.01 | 1.00 |
| 2.sex | 0.99 | 0.99 | 3.40 | 0.70 |
| 1.surgery#2.seerstage | 0.20 | 0.20 | 1.20 | 0.92 |
| 1.surgery#3.seerstage | 0.04 | 0.04 | <0.01 | 1.00 |
| 1.surgery#4.seerstage | 0.06 | 0.06 | <0.01 | 1.00 |
| c.age#c.age | 4905.50 | 4905.60 | <0.01 | 1.00 |
| 2.category#2.seerstage | 0.06 | 0.06 | 1.90 | 0.85 |
| 2.category#3.seerstage | 0.01 | 0.01 | <0.01 | 1.00 |
| 3.category#2.seerstage | 0.03 | 0.03 | <0.01 | 1.00 |
| 3.category#3.seerstage | 0.02 | 0.02 | <0.01 | 1.00 |
| 2.race#2.seerstage | 0.01 | 0.01 | <0.01 | 1.00 |
| 2.race#3.seerstage | 0.02 | 0.02 | <0.01 | 1.00 |
| 2.race#4.seerstage | 0.02 | 0.02 | 3.10 | 0.76 |
| 3.race#2.seerstage | 0.01 | 0.01 | <0.01 | 1.00 |
| 4.race#2.seerstage | 0.01 | 0.01 | <0.01 | 1.00 |
| 4.race#3.seerstage | 0.01 | 0.01 | <0.01 | 1.00 |
| 5.race#2.seerstage | <0.01 | <0.01 | <0.01 | 1.00 |
| 2.category#1.surgery | 0.25 | 0.25 | 0.80 | 0.92 |
| 2.category#2.surgery | <0.01 | <0.01 | <0.01 | 1.00 |
| 3.category#1.surgery | 0.13 | 0.13 | <0.01 | 1.00 |
| 2.category#2.race | 0.03 | 0.03 | <0.01 | 1.00 |
| 2.category#3.race | 0.01 | 0.01 | 2.70 | 0.70 |
| 2.category#4.race | 0.01 | 0.01 | <0.01 | 1.00 |
| 2.category#5.race | 0.01 | 0.01 | <0.01 | 1.00 |
| 3.category#2.race | 0.02 | 0.02 | <0.01 | 1.00 |
| 2.sex#2.seerstage | 0.20 | 0.20 | <0.01 | 1.00 |
| 2.sex#3.seerstage | 0.07 | 0.07 | 1.90 | 0.87 |
| 2.sex#4.seerstage | 0.08 | 0.08 | 1.50 | 0.88 |
| 2.race#1.radiation | 0.05 | 0.05 | 1.70 | 0.84 |
| 2.race#2.radiation | 0.08 | 0.08 | <0.01 | 1.00 |
| 3.race#1.radiation | 0.03 | 0.03 | 2.10 | 0.79 |
| 3.race#2.radiation | 0.01 | 0.01 | <0.01 | 1.00 |
| 4.race#1.radiation | 0.01 | 0.01 | <0.01 | 1.00 |
| 4.race#2.radiation | 0.02 | 0.02 | <0.01 | 1.00 |
| 5.race#2.radiation | 0.01 | 0.01 | <0.01 | 1.00 |
| 1.surgery#2.race | 0.11 | 0.11 | <0.01 | 1.00 |
| 1.surgery#3.race | 0.03 | 0.03 | <0.01 | 1.00 |
| 1.surgery#4.race | 0.02 | 0.02 | <0.01 | 1.00 |
| 1.surgery#5.race | 0.01 | 0.01 | <0.01 | 1.00 |
| 2.race#c.age | 8.95 | 8.74 | 1.00 | 0.91 |
| 3.race#c.age | 2.58 | 2.82 | 1.70 | 0.84 |
| 4.race#c.age | 2.06 | 2.06 | <0.01 | 1.00 |
| 5.race#c.age | 0.46 | 0.48 | 0.60 | 0.96 |
| 2.sex#2.race | 0.14 | 0.13 | 1.10 | 0.90 |
| 2.sex#3.race | 0.04 | 0.04 | 1.70 | 0.82 |
| 2.sex#4.race | 0.03 | 0.03 | <0.01 | 1.00 |
| 2.sex#5.race | 0.01 | 0.01 | <0.01 | 1.00 |
| 2.category#c.age | 18.72 | 18.64 | 0.30 | 0.98 |
| 3.category#c.age | 8.97 | 8.97 | <0.01 | 1.00 |

LABC: breast duct carcinoma with prior primary lung adenocarcinoma; bias: absolutely standardized difference; p: t test between treated and control group

Tab S23 Balance test between group mTPC2 with LABC and single breast cancer in matched cohort

|  | Mean | |  |  |
| --- | --- | --- | --- | --- |
| Variable | Treated | Control | bias | p |
| age | 73.12 | 73.33 | -1.80 | 0.84 |
| 2.seerstage | 0.17 | 0.17 | <0.01 | 1.00 |
| 3.seerstage | 0.06 | 0.06 | -3.60 | 0.81 |
| 4.seerstage | 0.14 | 0.13 | 2.10 | 0.87 |
| 1.radiation | 0.53 | 0.53 | 1.20 | 0.91 |
| 2.radiation | 0.47 | 0.48 | -1.20 | 0.91 |
| 1.surgery | 0.93 | 0.93 | <0.01 | 1.00 |
| 2.race | 0.11 | 0.11 | <0.01 | 1.00 |
| 3.race | 0.04 | 0.04 | -3.10 | 0.78 |
| 4.race | 0.04 | 0.04 | <0.01 | 1.00 |
| 5.race | 0.01 | 0.01 | <0.01 | 1.00 |
| 2.category | 0.25 | 0.25 | <0.01 | 1.00 |
| 3.category | 0.13 | 0.14 | -1.70 | 0.87 |
| 1.marital | 0.43 | 0.42 | 1.30 | 0.91 |
| 2.marital | 0.07 | 0.05 | 8.40 | 0.48 |
| 2.category#2.seerstage | 0.06 | 0.06 | <0.01 | 1.00 |
| 2.category#3.seerstage | 0.01 | 0.01 | <0.01 | 1.00 |
| 3.category#2.seerstage | 0.04 | 0.04 | <0.01 | 1.00 |
| 3.category#3.seerstage | 0.03 | 0.03 | -5.30 | 0.74 |
| c.age#c.age | 5430.60 | 5462.10 | -2.00 | 0.83 |
| 1.surgery#2.seerstage | 0.17 | 0.17 | <0.01 | 1.00 |
| 1.surgery#3.seerstage | 0.03 | 0.04 | -4.70 | 0.76 |
| 1.surgery#4.seerstage | 0.12 | 0.11 | 2.20 | 0.86 |
| 1.marital#2.seerstage | 0.07 | 0.06 | 3.20 | 0.82 |
| 1.marital#3.seerstage | 0.02 | 0.02 | <0.01 | 1.00 |
| 1.marital#4.seerstage | 0.05 | 0.05 | <0.01 | 1.00 |
| 2.marital#2.seerstage | 0.01 | 0.01 | <0.01 | 1.00 |
| 2.marital#4.seerstage | 0.03 | 0.01 | 16.20 | 0.18 |
| 1.marital#2.race | 0.01 | <0.01 | 4.30 | 0.32 |
| 1.marital#3.race | 0.02 | 0.02 | <0.01 | 1.00 |
| 1.marital#4.race | 0.03 | 0.03 | <0.01 | 1.00 |
| 1.marital#5.race | 0.01 | 0.01 | <0.01 | 1.00 |
| 2.marital#2.race | 0.01 | 0.01 | 6.70 | 0.56 |
| 2.marital#4.race | 0.01 | 0.01 | <0.01 | 1.00 |
| 2.race#2.seerstage | 0.04 | 0.03 | 4.40 | 0.76 |
| 2.race#3.seerstage | 0.01 | 0.01 | -10.20 | 0.56 |
| 2.race#4.seerstage | 0.03 | 0.03 | <0.01 | 1.00 |
| 3.race#2.seerstage | 0.01 | 0.01 | -10.30 | 0.56 |
| 3.race#4.seerstage | 0.01 | 0.01 | <0.01 | 1.00 |
| 4.race#4.seerstage | 0.01 | 0.01 | <0.01 | 1.00 |
| 2.category#2.race | 0.02 | 0.02 | <0.01 | 1.00 |
| 2.category#4.race | 0.01 | 0.01 | <0.01 | 1.00 |
| 3.category#2.race | 0.01 | 0.01 | <0.01 | 1.00 |
| 3.category#4.race | 0.01 | 0.01 | <0.01 | 1.00 |
| 2.race#1.radiation | 0.06 | 0.06 | <0.01 | 1.00 |
| 2.race#2.radiation | 0.04 | 0.04 | <0.01 | 1.00 |
| 3.race#1.radiation | 0.02 | 0.02 | <0.01 | 1.00 |
| 3.race#2.radiation | 0.02 | 0.03 | -4.40 | 0.70 |
| 4.race#1.radiation | 0.01 | 0.01 | <0.01 | 1.00 |
| 4.race#2.radiation | 0.04 | 0.04 | <0.01 | 1.00 |
| 5.race#1.radiation | 0.01 | 0.01 | <0.01 | 1.00 |
| 2.race#c.age | 7.76 | 7.82 | -0.30 | 0.98 |
| 3.race#c.age | 2.65 | 3.20 | -4.30 | 0.73 |
| 4.race#c.age | 3.04 | 3.04 | <0.01 | 1.00 |
| 5.race#c.age | 0.51 | 0.51 | <0.01 | 1.00 |
| 2.category#c.age | 18.11 | 18.11 | <0.01 | 1.00 |
| 3.category#c.age | 9.34 | 9.77 | -1.70 | 0.88 |
| 1.surgery#1.radiation | 0.51 | 0.50 | 1.20 | 0.91 |
| 1.surgery#2.radiation | 0.43 | 0.43 | -1.30 | 0.91 |
| 1.marital#2.category | 0.09 | 0.09 | <0.01 | 1.00 |
| 1.marital#3.category | 0.06 | 0.07 | -2.30 | 0.82 |
| 2.marital#2.category | 0.03 | 0.03 | <0.01 | 1.00 |
| 2.marital#3.category | 0.01 | 0.01 | <0.01 | 1.00 |
| 1.surgery#c.age | 68.14 | 68.31 | -0.90 | 0.94 |
| 2.race#1.surgery | 0.09 | 0.09 | 2.10 | 0.85 |
| 3.race#1.surgery | 0.04 | 0.04 | -3.10 | 0.78 |
| 4.race#1.surgery | 0.04 | 0.04 | <0.01 | 1.00 |
| 5.race#1.surgery | 0.01 | 0.01 | <0.01 | 1.00 |

LABC: breast duct carcinoma with prior primary lung adenocarcinoma; bias: absolutely standardized difference; p: t test between treated and control group
